# Supplementary material for: Enhanced energy storage in high-entropy superparaelectrics via local ferroelectric polarization
Source: Nat Commun. 2026 Apr 8;17:4999. doi: 10.1038/s41467-026-71370-7 (PMC13237357; doi:10.1038/s41467-026-71370-7)
Supplement: Supplementary file 1 — Supplementary Information [file 41467_2026_71370_MOESM1_ESM.pdf]

Supplementary Information

Enhanced Energy Storage in High-Entropy Superparaelectric via Local Ferroelectric Polarization

Tongxin Wei [1], Jinzhu Zou [1]*, Miao Song[1], Kai Zhu[1], Zhongna, Yan [2], Zhifang Zhou[1], Xuefan Zhou[1], Kechao Zhou[1], Shujun Zhang [3]* & Dou Zhang [1]*

[1] State Key Laboratory of Powder Metallurgy, Central South University, Changsha, Hunan 410083, China.

[2] School of Energy and Power Engineering, Changsha University of Science and Technology, Changsha, Hunan 410083, China.

[3] Department of Chemistry, City University of Hong Kong, Kowloon, 999077, Hong Kong, China.

*Corresponding authors.

E-mail address: jinzhuzou@csu.edu.cn; s.j.zhang@cityu.edu.hk; dzhang@csu.edu.cn

Supplementary Note 1: Phase field simulations

Phase field simulations were performed to show the impact of elemental properties on the ferroelectric domain structure evolution, and therefore, on the polarization response. In the phase-field model, time-dependent Ginzburg-Landau equation was used to describe the evolution of the polarization $\boldsymbol{P}(x, t)$ for the ferroelectric system, as follows [1]:

$$\frac{\partial \boldsymbol{P}}{\partial t} = -L\frac{\delta F}{\delta \boldsymbol{P}} \tag{1}$$

where x and t are the space position and time, respectively, L is the kinetic coefficient, and F means the total free energy of the system. The above equation describes that the polarization will evolve over time in the direction of minimizing the free energy of the system. The total free energy can be expressed as follows:

$$F = \int_V f_{bulk} + f_{elas} + f_{elec} + f_{grad}\, dV \tag{2}$$

where V denotes the system volume, f_{bulk} the Landau bulk free energy density, f_{elas} the elastic energy density, f_{elec} the electrostatic energy density and f_{grad} the gradient energy density[2]. To simulate the response of dielectric materials under an external electric field, an alternating current (AC) electric field was applied to the simulation region in order to obtain P–E loops and analyze the behavior of different polar entities in the superparaelectric state (as shown in Figs. S1a–b).

We adopted a finite element method to numerically solve the above partial differential equations[3-5]. The triangular elements were used for the space discretization. Specifically, it is performed through *the mathematical interface* in the *COMSOL* software due to its powerful solving capabilities in Multiphysics problems.

1.1 Polarization field of high entropy superparaelectric

We used previously reported Landau coefficients for ferroelectric materials as the initial state[6]. To simulate the effect of introducing a high-entropy strategy on domain structures (constructing a superparaelectric state), we employed the method developed by Yang[7], treating multiple elements as point defects randomly distributed within the ferroelectric matrix (Figs. S2a1–a2). As illustrated in Figs. S2b1-b2, the Landau coefficients were modified accordingly (T_c - c*657). As shown in Figs. S2c1–c2, for simulations incorporating the high-entropy strategy, the domain structure is refined into polar nanoregions, corresponding to its superparaelectric state structure. In contrast, the simulation domain without introduced point defects exhibits a clear long-range ordered structure. In subsequent simulations based on this setup, we respectively configured paraelectric, antiferroelectric, and ferroelectric phases within the superparaelectric simulation region, with their Landau coefficients set based on Reference[8].

1.2 Phase-field simulation of different ABO_3 constituents in a superparaelectric matrix

As shown in Fig. S3a, to simulate the influence of ferroelectric, paraelectric, and antiferroelectric constituents on polarization in a high-entropy superparaelectric matrix, based on the setup in Section 1.1, we assigned Landau coefficients corresponding to paraelectric, antiferroelectric, and ferroelectric states to local regions[8] and applied the same alternating electric field to analyze their P-E loops. Specifically, for the antiferroelectric state, the method by Huang et al. was employed to simulate its polarization response, and its Landau free energy curve is shown in Fig. S3b. As shown in Fig. S3c, for the ferroelectric state, parameters from M.J. Haun were used to simulate a strongly ferroelectric $PbTiO_3$[9]. For the paraelectric state, a single Landau coefficient was set for simulation, with its free energy curve presented in Fig. S3d. Figs. S3e–g display the P-E curves of the material systems corresponding to these Landau coefficients under an applied electric field, demonstrating that the above settings can effectively simulate the polarization response characteristics of antiferroelectric, ferroelectric, and paraelectric materials under an external electric field.

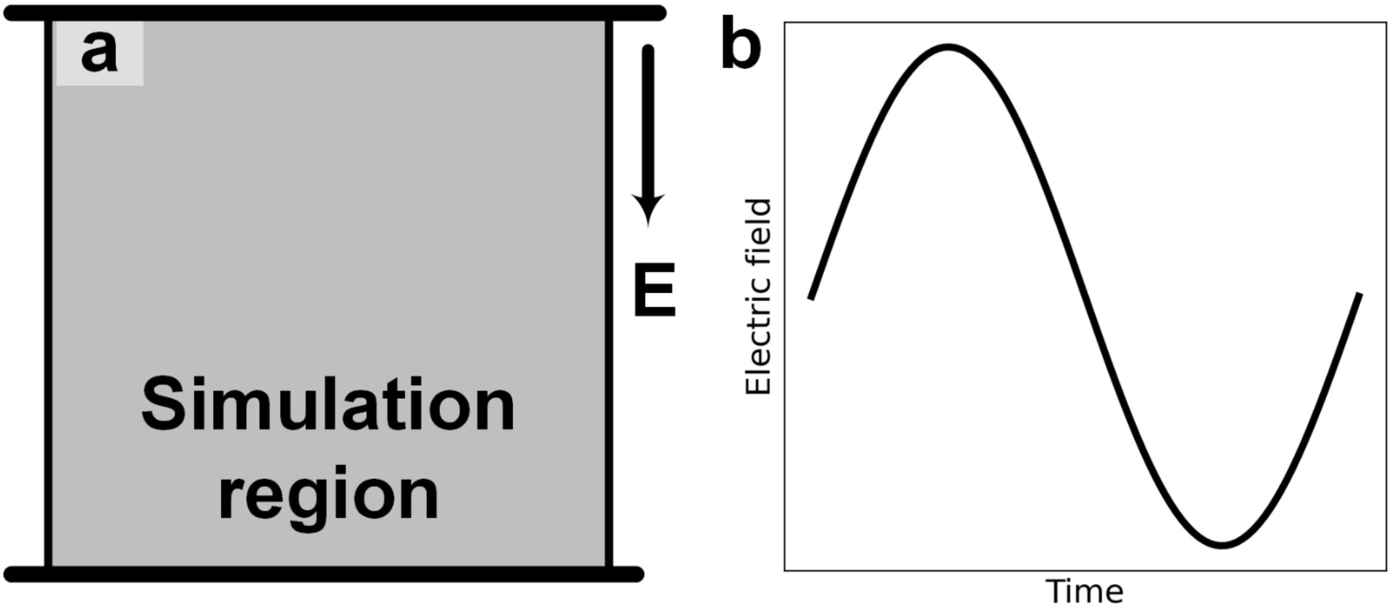

Figure S1. Schematic of the phase-field simulation. (a) Simulation region and direction of the applied electric field; (b) schematic of the applied sinusoidal electric field.

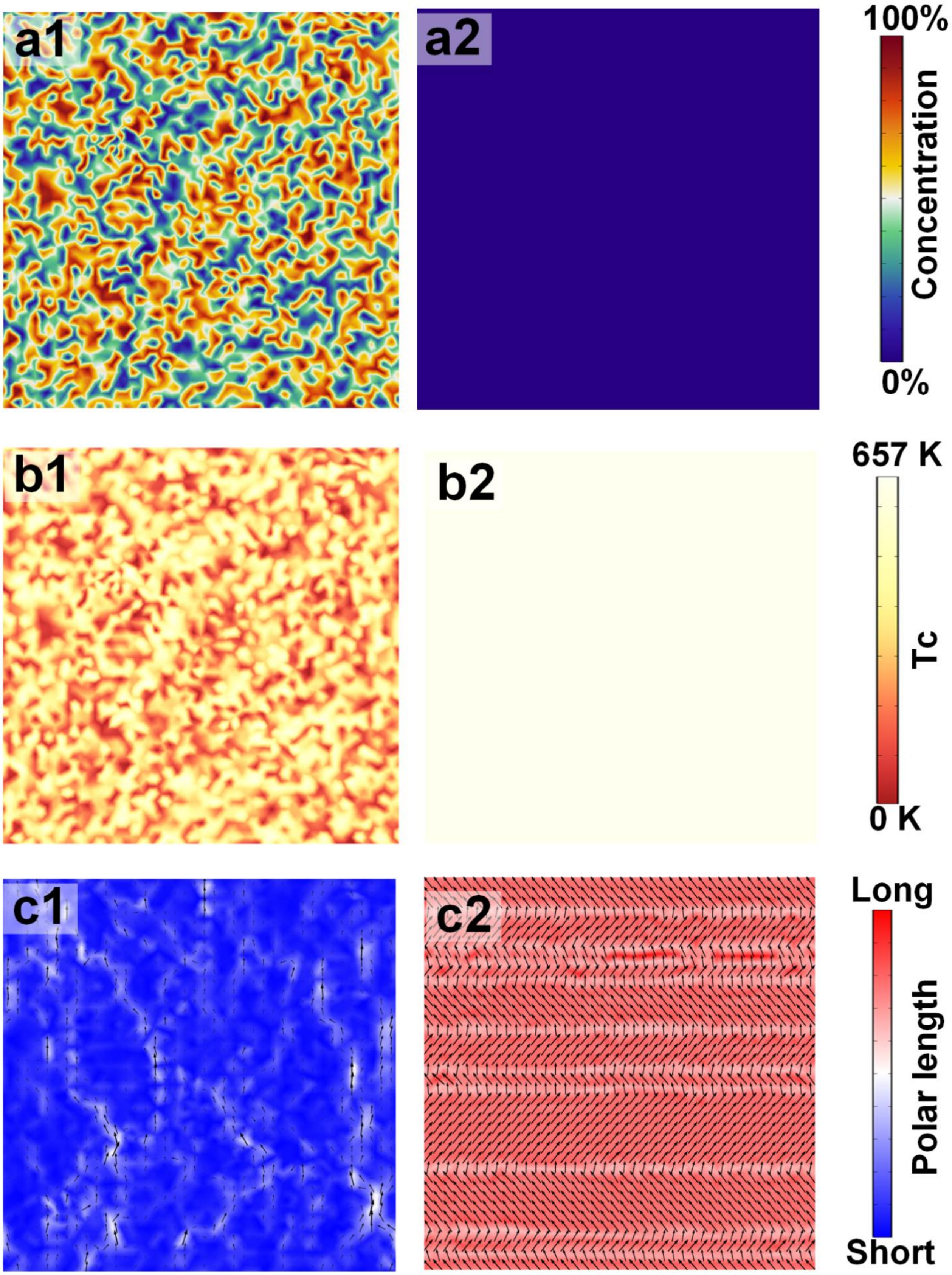

Figure S2. Phase-field simulation of the high-entropy strategy. (a1) Schematic of the chemical distribution for the high-entropy case; (a2) chemical distribution for the non-high-entropy case; (b1) Distribution of Tc after introducing the high-entropy strategy; (b2) Tc distribution for the non-high-entropy case; (c1) Polarization configuration under the high-entropy strategy, exhibiting a superparaelectric state structure; (c2) Polarization configuration for the non-high-entropy case, showing a long-range ordered structure.

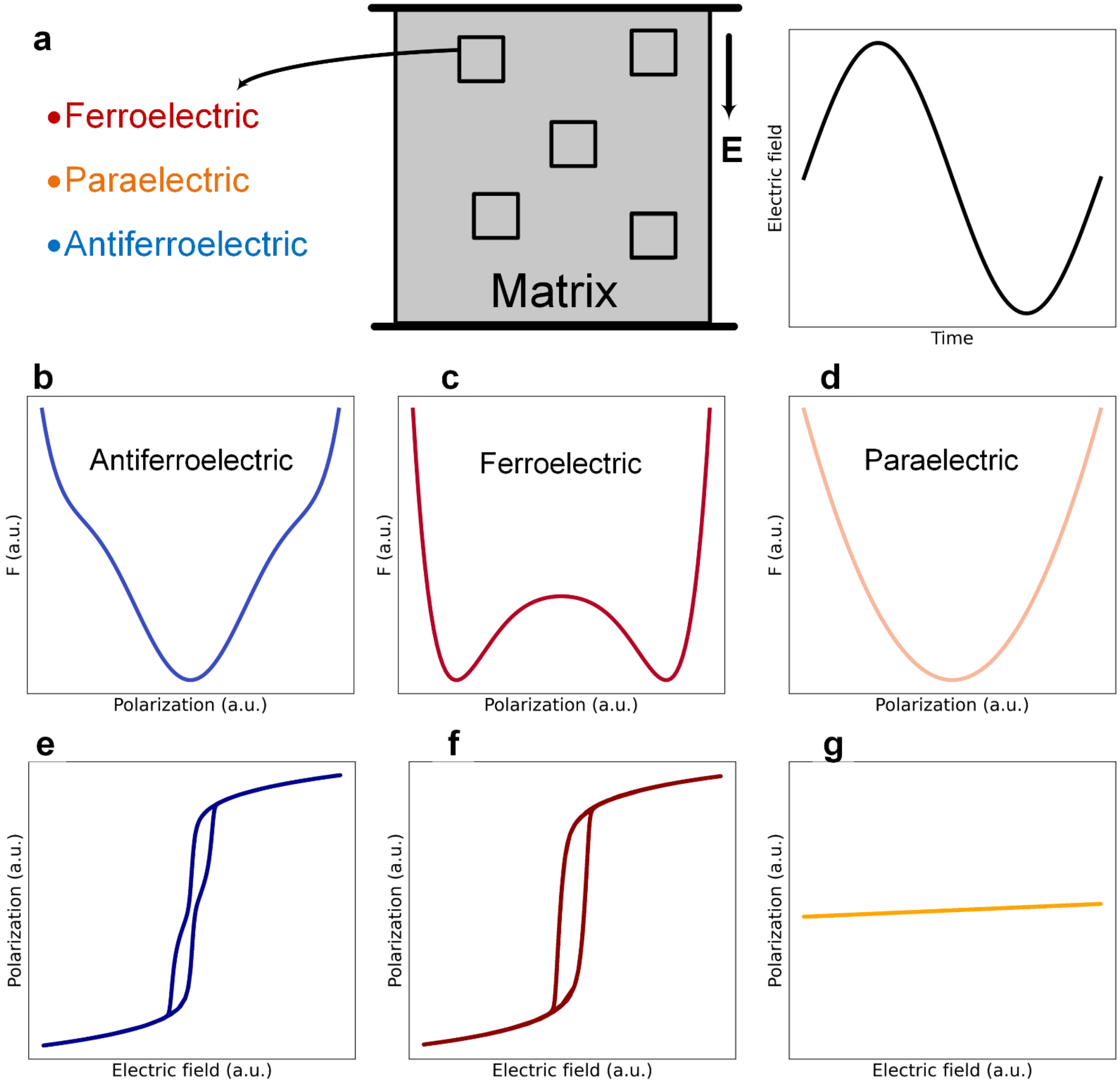

Figure S3. Phase-field simulation of different ABO_3 constituents in a superparaelectric matrix. (a) Simulation setup, in which Landau coefficients are assigned to local regions (squares) within a superparaelectric matrix to simulate the polarization responses of ferroelectric, paraelectric, and antiferroelectric phases; Landau free energy curves for the (b) antiferroelectric, (c) ferroelectric, and (d) paraelectric phases; (e)–(g) simulated P–E loops for the (e) antiferroelectric, (f) ferroelectric, and (g) paraelectric phases based on the corresponding Landau coefficient settings.

Supplementary Note 2: Analysis of the Polarization Configuration and Switching Behavior of Strongly Ferroelectric Phases in a Superparaelectric Matrix

2.1 Evolution of Polarization Configuration

For ferroelectric materials that are not influenced by the surrounding superparaelectric polarization, their spontaneous polarization is governed by their intrinsic Landau energy curve (bulk free energy), resulting in a distinct long-range ordered structure (Figs. S4a). This leads to materials with high saturation polarization and high remanent polarization, as shown in Figs. S3f.

In contrast, when a local ferroelectric phase structure is introduced into a superparaelectric matrix, the polarization configuration within the ferroelectric region is influenced not only by its bulk free energy but also by the weakly polarized superparaelectric surroundings. As a result, the spontaneous polarization within the local ferroelectric region struggles to maintain long-range order. Instead, while the polarization magnitude remains relatively large, its orientation becomes disordered (Fig. S4b). This characteristic facilitates the attainment of high saturation polarization yet low remanent polarization in the material under an external electric field, as discussed below.

2.2 Analysis of Polarization Switching Behavior

In addition to changes in polarization alignment, the polarization switching behavior of ferroelectric phases within a superparaelectric matrix also differs significantly. As shown in Fig. S5a, considering a polarization state oriented by a positive electric field, the polarization gradually switches as the external electric field progressively increases in the negative direction. As illustrated in Fig. S5b, for a pure ferroelectric state, the rotation of spontaneous polarization occurs through exhibited an overall and uniform switching, a slow process that makes complete switching difficult within the same time step.

In contrast, as depicted in Fig. S5c, for the ferroelectric phase within the superparaelectric matrix, the polarization of the superparaelectric surroundings responds easily to the external field. Under the influence of gradient energy, the polarization of the ferroelectric phase can also switch rapidly, responding swiftly to the external field within the same time step. It is precisely due to this effect that introducing strong ferroelectric phases into a superparaelectric matrix is expected to enhance the maximum polarization while maintaining low remanent polarization, thereby improving energy storage density.

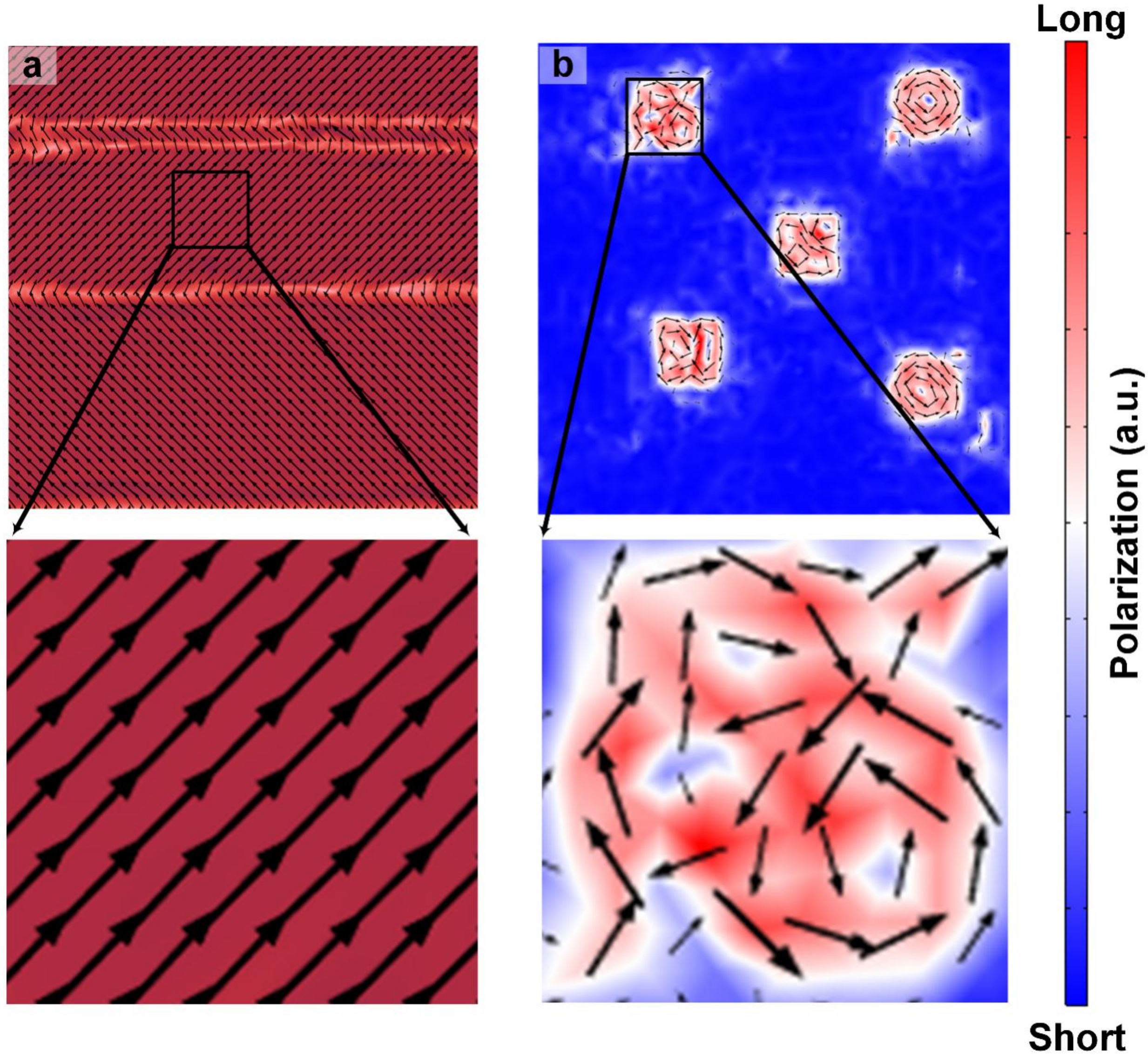

Figure S4 Polarization configurations of the ferroelectric phase: in its isolated state and within the superparaelectric matrix. (a) When influenced solely by its own bulk free energy (Landau energy), the ferroelectric phase exhibits long-range ordered polarization. (b) Within a superparaelectric matrix, the polarization directions in locally distributed ferroelectric phases become disordered due to the influence of the polarization from the neighboring superparaelectric matrix.

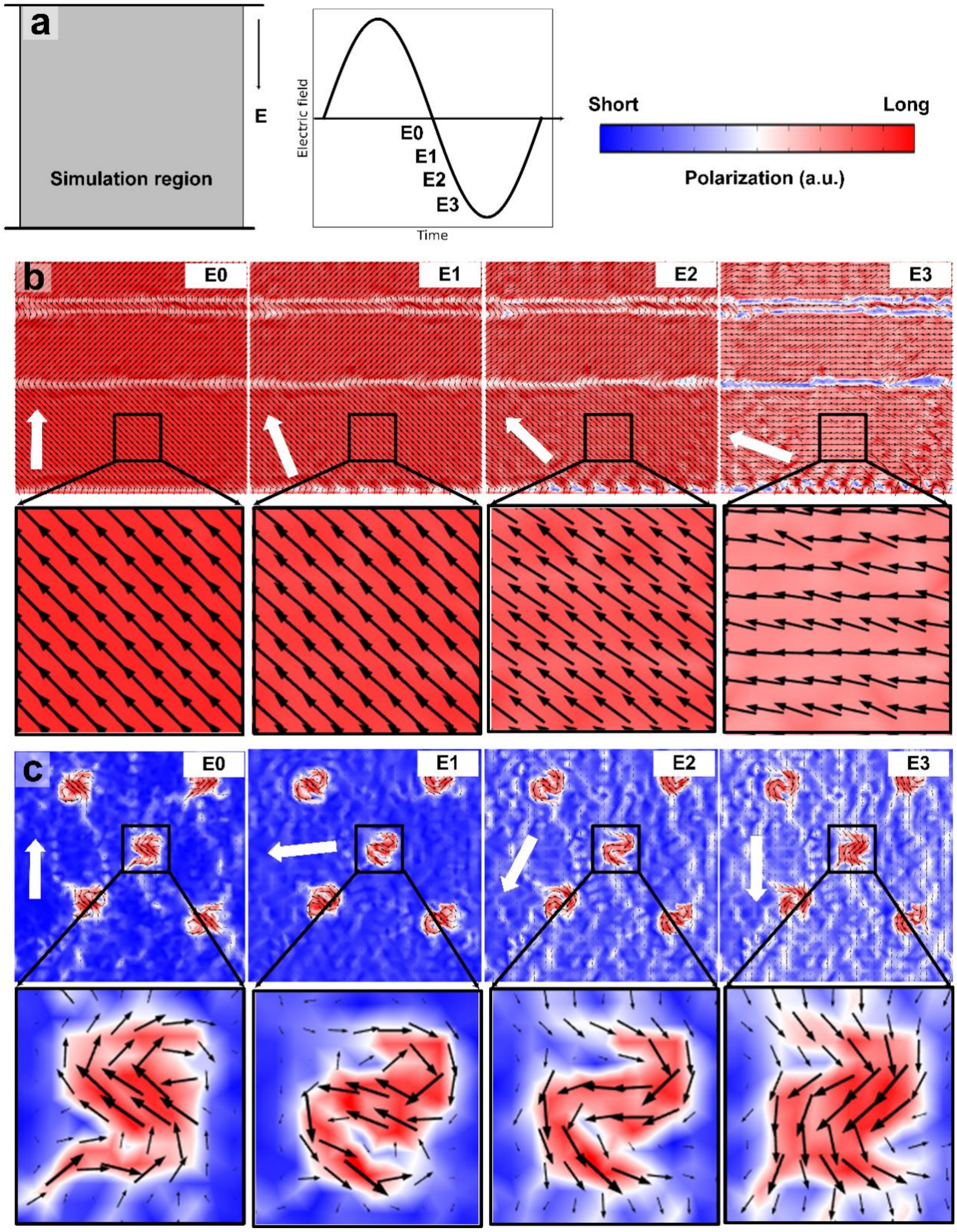

Figure S5. Analysis of polarization switching in the ferroelectric phase itself and under the influence of the superparaelectric state. (a) Schematic of the simulation: a sinusoidal electric field is applied to the simulation region to analyze polarization switching behavior, with results at time steps E0, E1, E2, and E3 visualized in panels (b) and (c). As the negative electric field is applied, the polarization gradually switches downward. (b) Polarization switching under the influence of the Landau energy of the ferroelectric phase itself: the white arrow indicates the overall polarization direction of the entire simulation region, showing that polarization switching is relatively difficult at the same time step. (c) Switching behavior of the ferroelectric phase influenced by the superparaelectric matrix: the white arrow indicates the overall polarization direction of the entire simulation region, demonstrating rapid polarization switching at the same time step.

Supplementary Note 3: DFT calculations for $CaTiO_3$, $BaTiO_3$, $PbTiO_3$ and $PbZrO_3$

DFT calculations were performed by using the Vienna ab-initio simulation package (VASP)[10]. For the exchange-correlation functional, the generalized gradient approximation (GGA) of Perdew-Burke-Emzerhof for solid was used[11]. Electron-ion interactions were described by the projector augmented-wave (PAW) potential with a kinetic energy cut-off of 500 eV. The Pb 5d6s6p, Ba 5s5p6s, Ti 3d4s, Zr 4s4p5s4d, Ca 3p4s and O 2s2p states were treated as valence electrons. The Brillouin zone integrations were sampled by using 4 × 4 × 4 Monkhorst-Pack grids for all the calculations. The energy convergence criterion was set to be 10^{-6} eV and the structure was fully relaxed until the force was <0.02 eV/Å. After the completion of the structural optimization, further calculations of charge density and density of state were conducted.

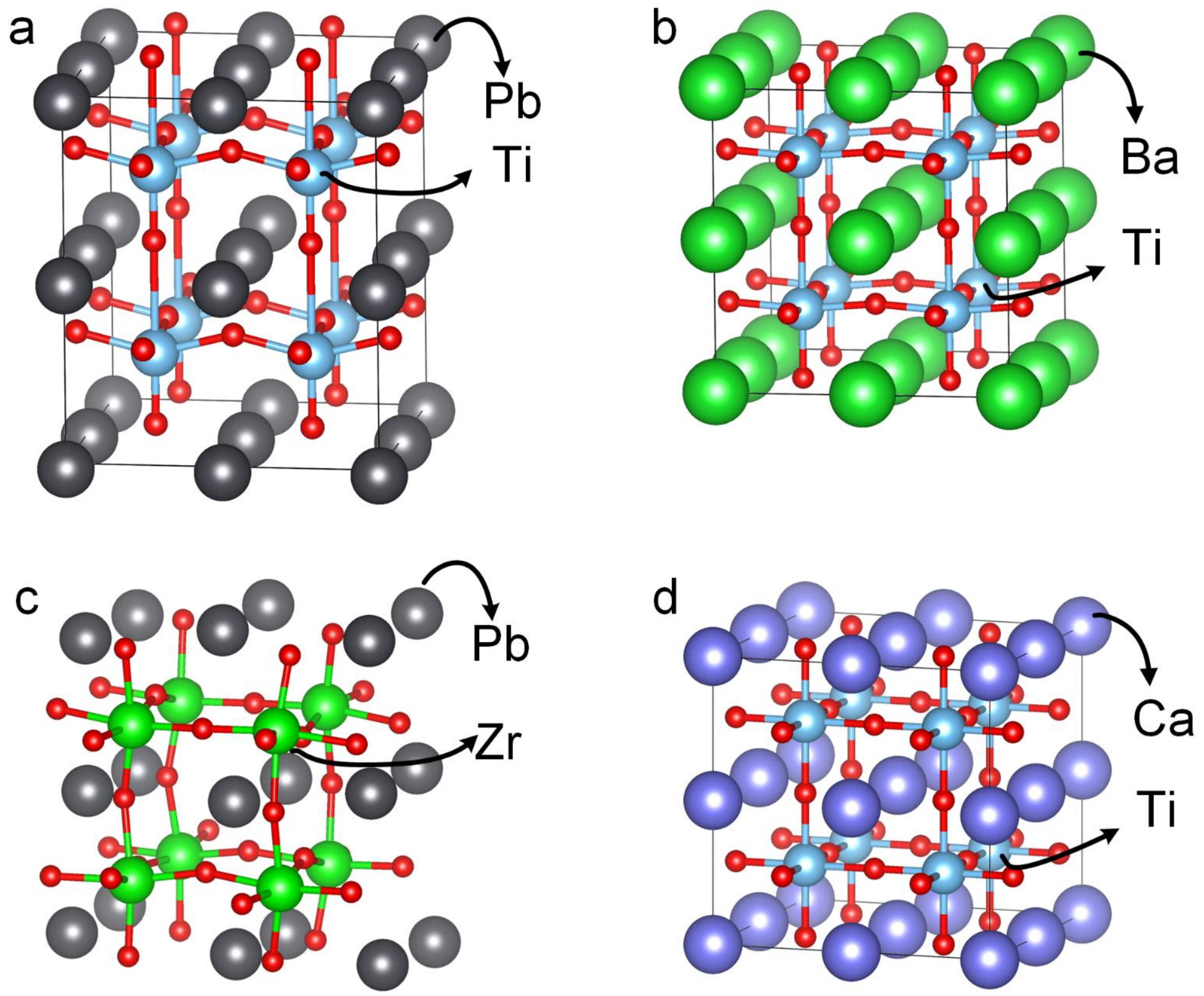

Figure S6 2*2*2 supercells used for DFT calculations. (a) $PbTiO_3$, (b) $BaTiO_3$, (c) $PbZrO_3$ and (d) $CaTiO_3$.

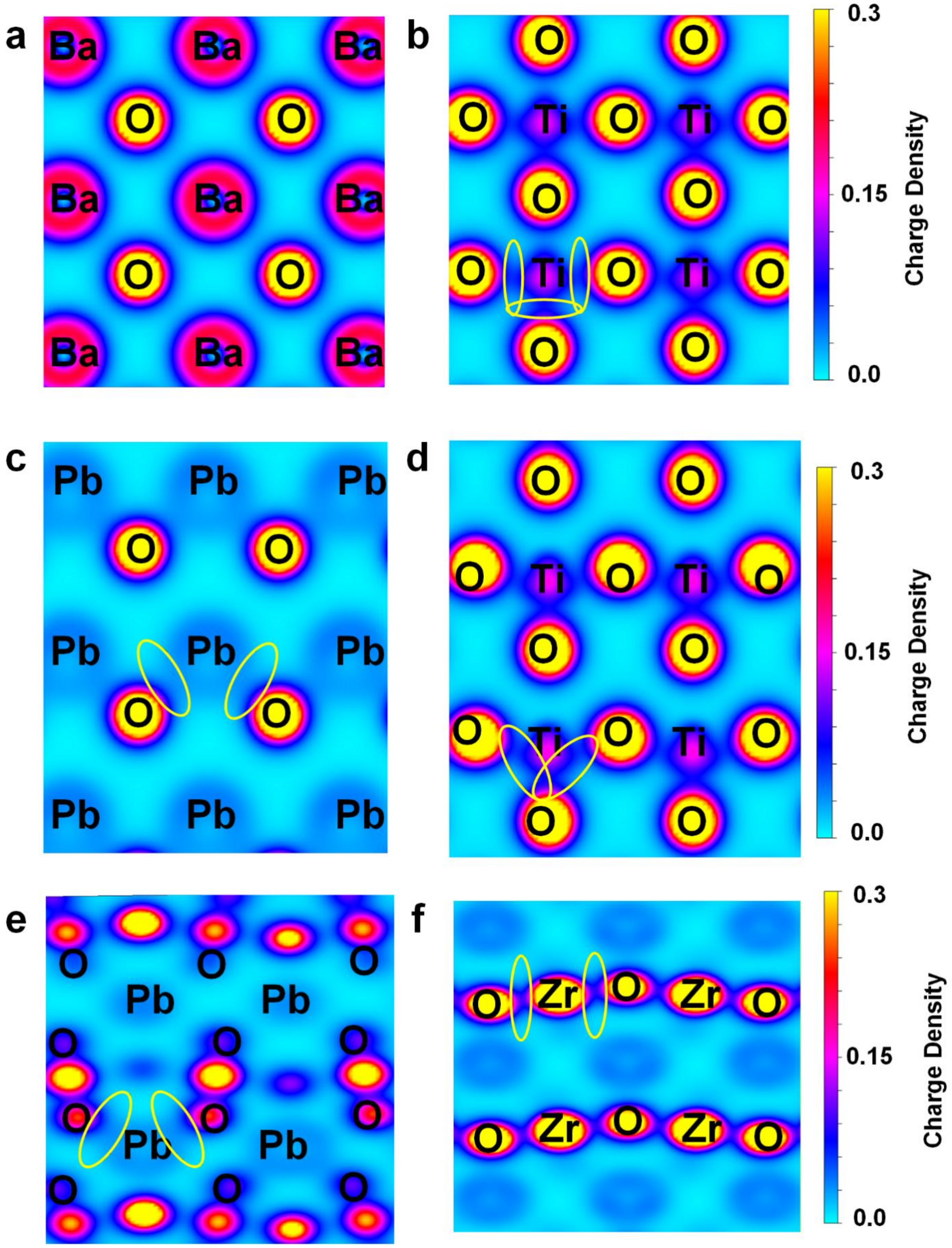

Figure S7 Charge Density. (a) Ba-O in $BaTiO_3$; (b) Ti-O in $BaTiO_3$; (c) Pb-O in $PbTiO_3$; (d) Ti-O in $PbTiO_3$; (e) Pb-O in $PbZrO_3$ and (f) Zr-O in $PbZrO_3$

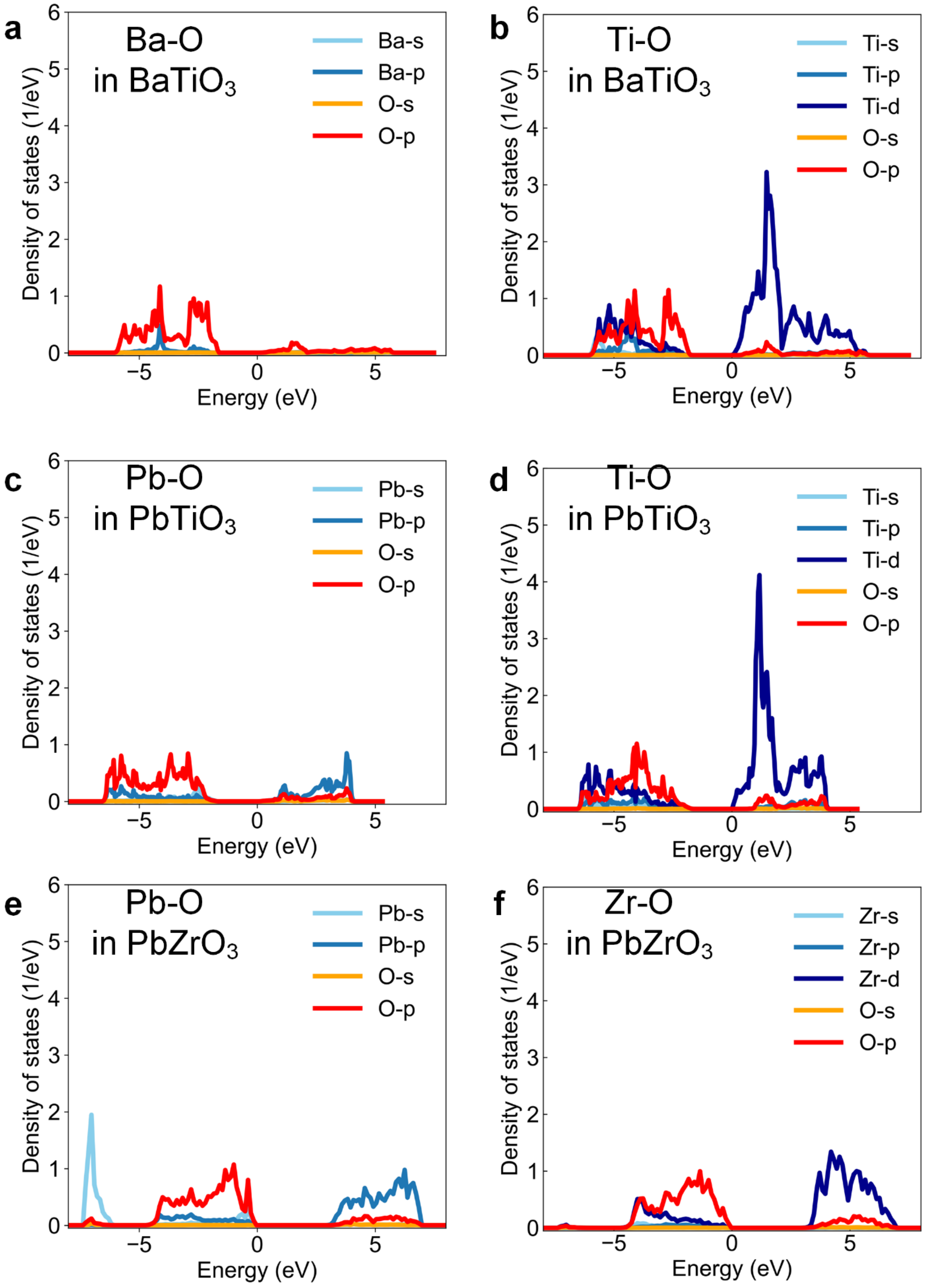

Figure S8 Density of states. (a) Ba-O in $BaTiO_3$; (b) Ti-O in $BaTiO_3$; (c) Pb-O in $PbTiO_3$; (d) Ti-O in $PbTiO_3$; (e) Pb-O in $PbZrO_3$ and (f) Zr-O in $PbZrO_3$

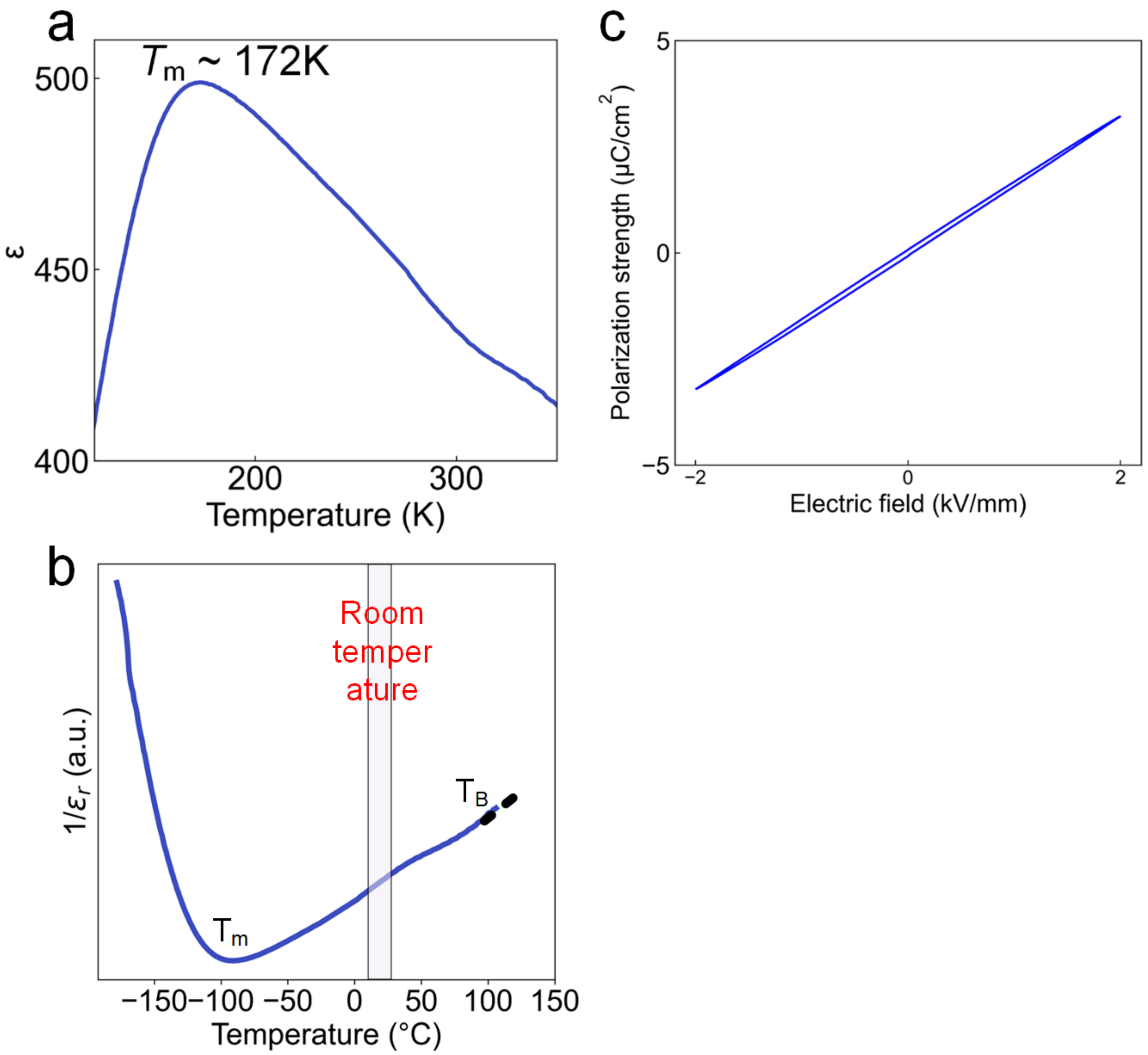

Figure S9 Dielectric and Ferroelectric Properties of BNKLSTZ. Temperature-dependent (a) permittivity and (b) $1/\varepsilon_r$ for BNKLSTZ at 1kHz; (c) P-E hysteresis loops of BNKLSTZ at 2kV/mm

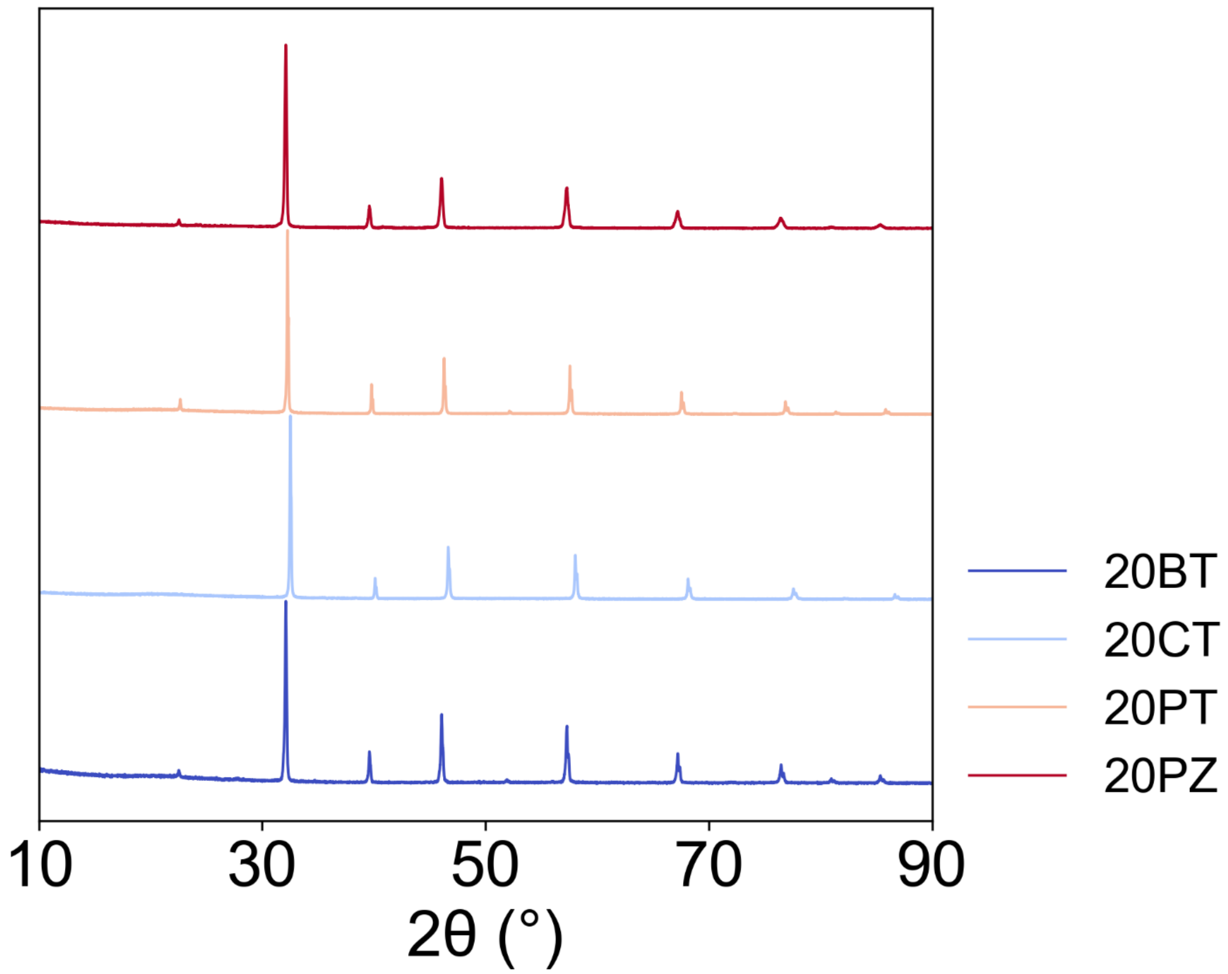

S10 XRD patterns of 20BT, 20CT, 20PT and 20PZ modified BNKLSTZ.

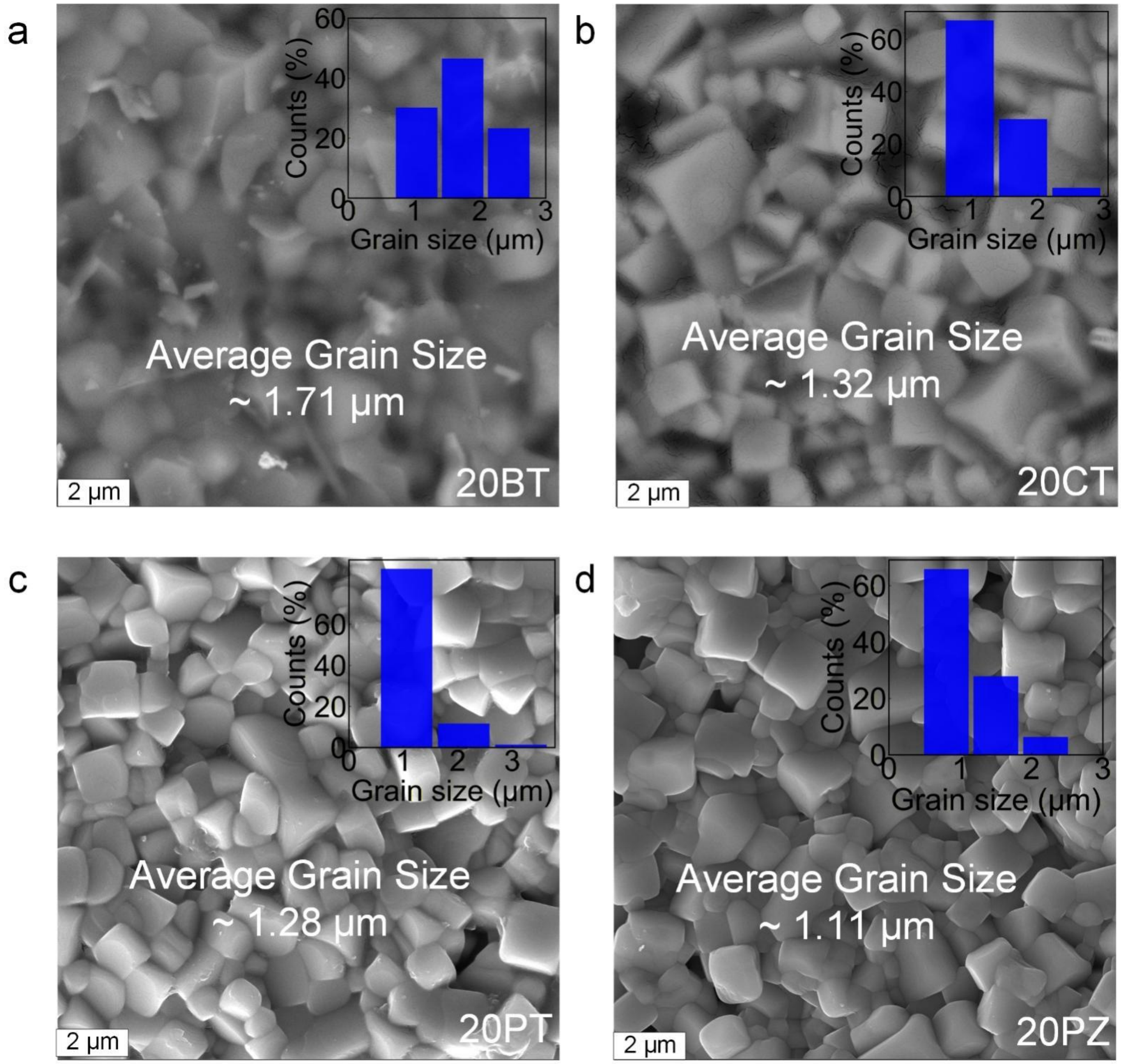

Figure S11 SEM observations of BNKLSTZ-based superparaelectric ceramics with different ferroelectric components introduced: (a) 20%$BaTiO_3$; (b) 20%$CaTiO_3$; (c) 20%$PbTiO_3$; (d) 20%$PbZrO_3$.

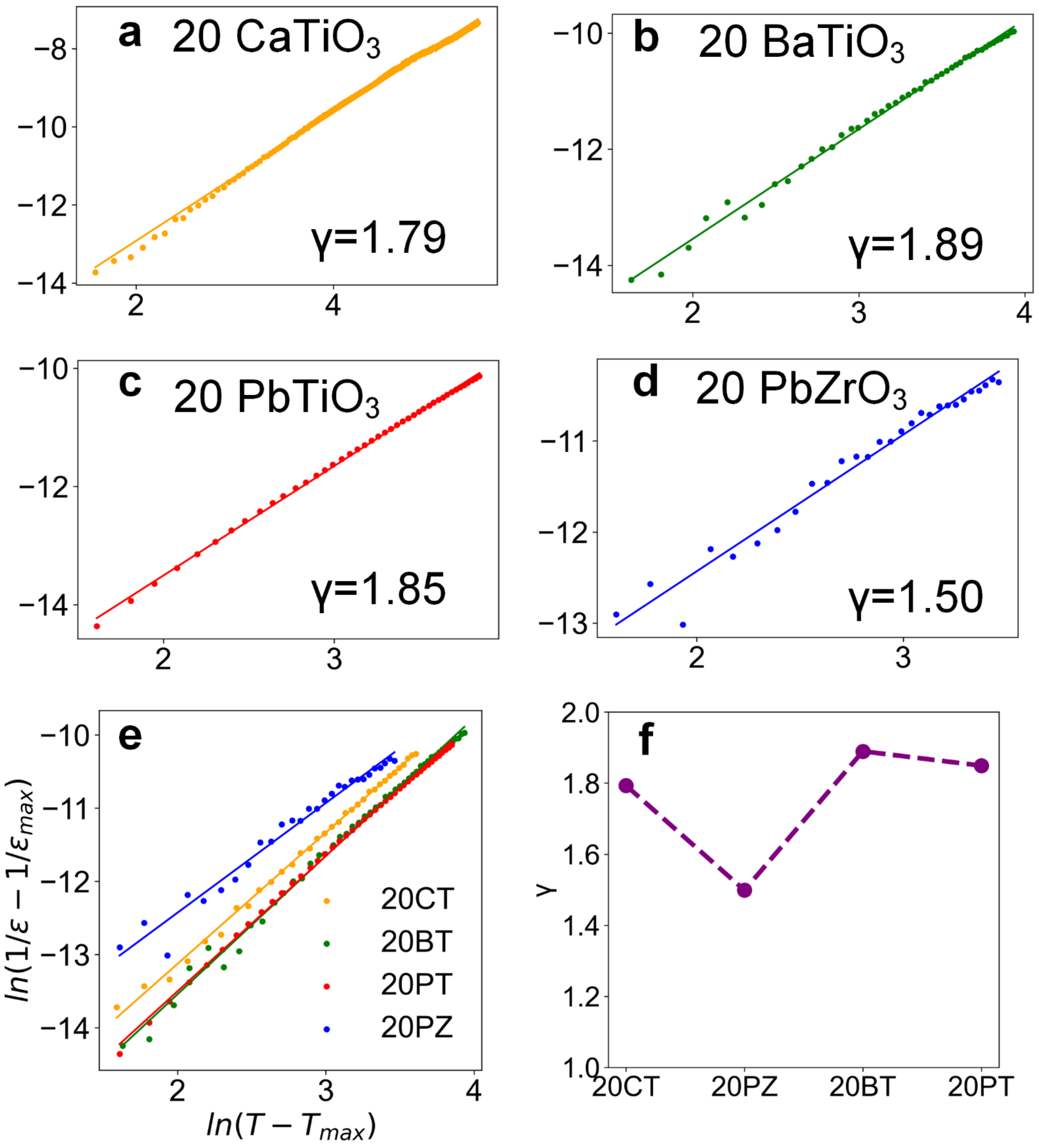

Figure S12 Relaxor degree fitting by modified Curie-Weiss law of BNKLSTZ-based superparaelectric ceramics with different components introduced. (a) 20%$CaTiO_3$; (b) 20%$BaTiO_3$; (c) 20%$PbTiO_3$; (d) 20%$PbZrO_3$; (e) Comparison of the fitting results and (f) corresponding relaxor degree γ.

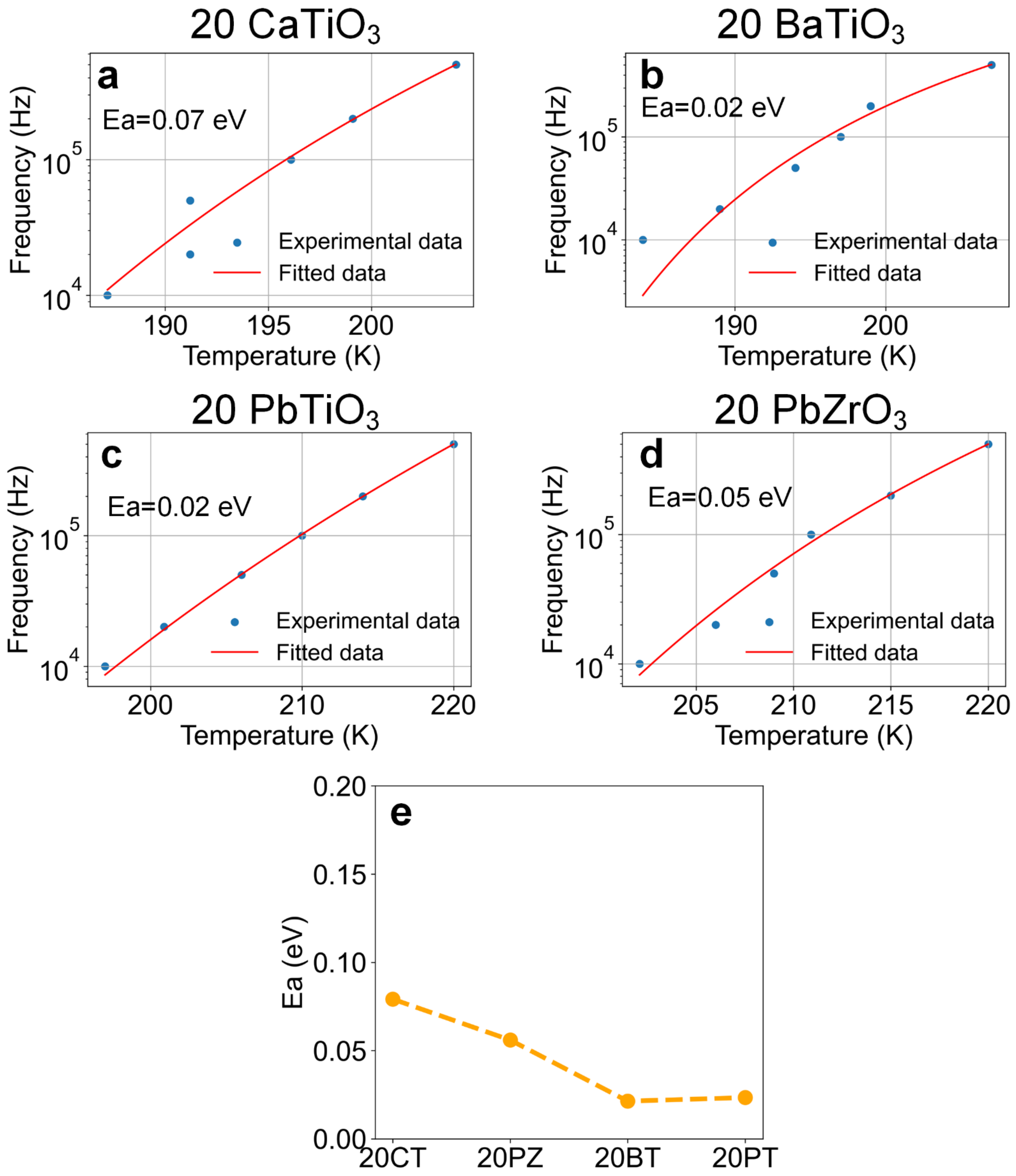

Figure S13 Vogel-Fulcher fitting of BNKLSTZ-based superparaelectric ceramics with different components introduced. (a) 20%$CaTiO_3$; (b) 20%$BaTiO_3$; (c) 20%$PbTiO_3$; (d) 20%$PbZrO_3$; (e) Comparison of E_a.

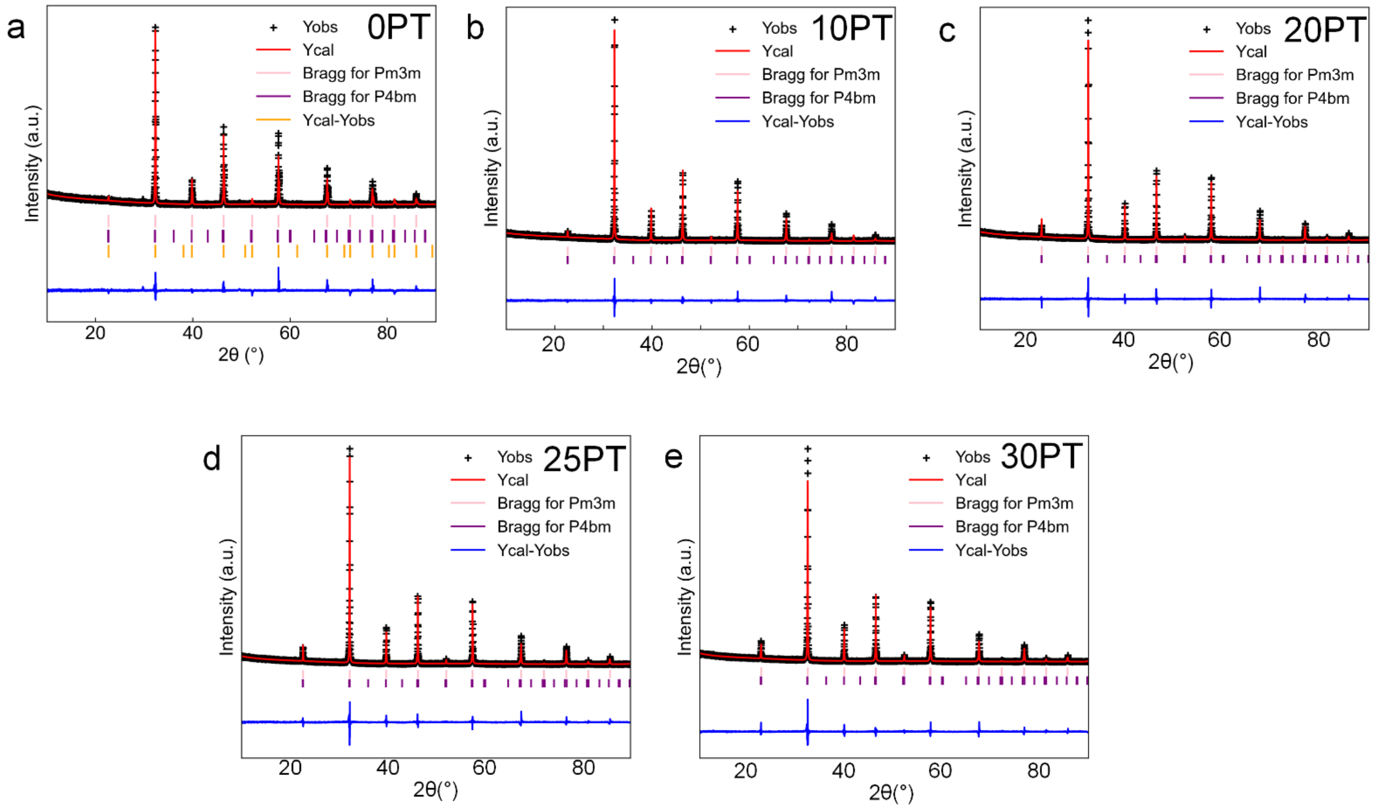

Figure S14 Rietveld refinement of XRD patterns for BNKLSTZ with varying $PbTiO_3$ contents x. (a) x = 0; (b) x = 0.1; (c) x = 0.2; (d) x = 0.25; (e) x = 0.3

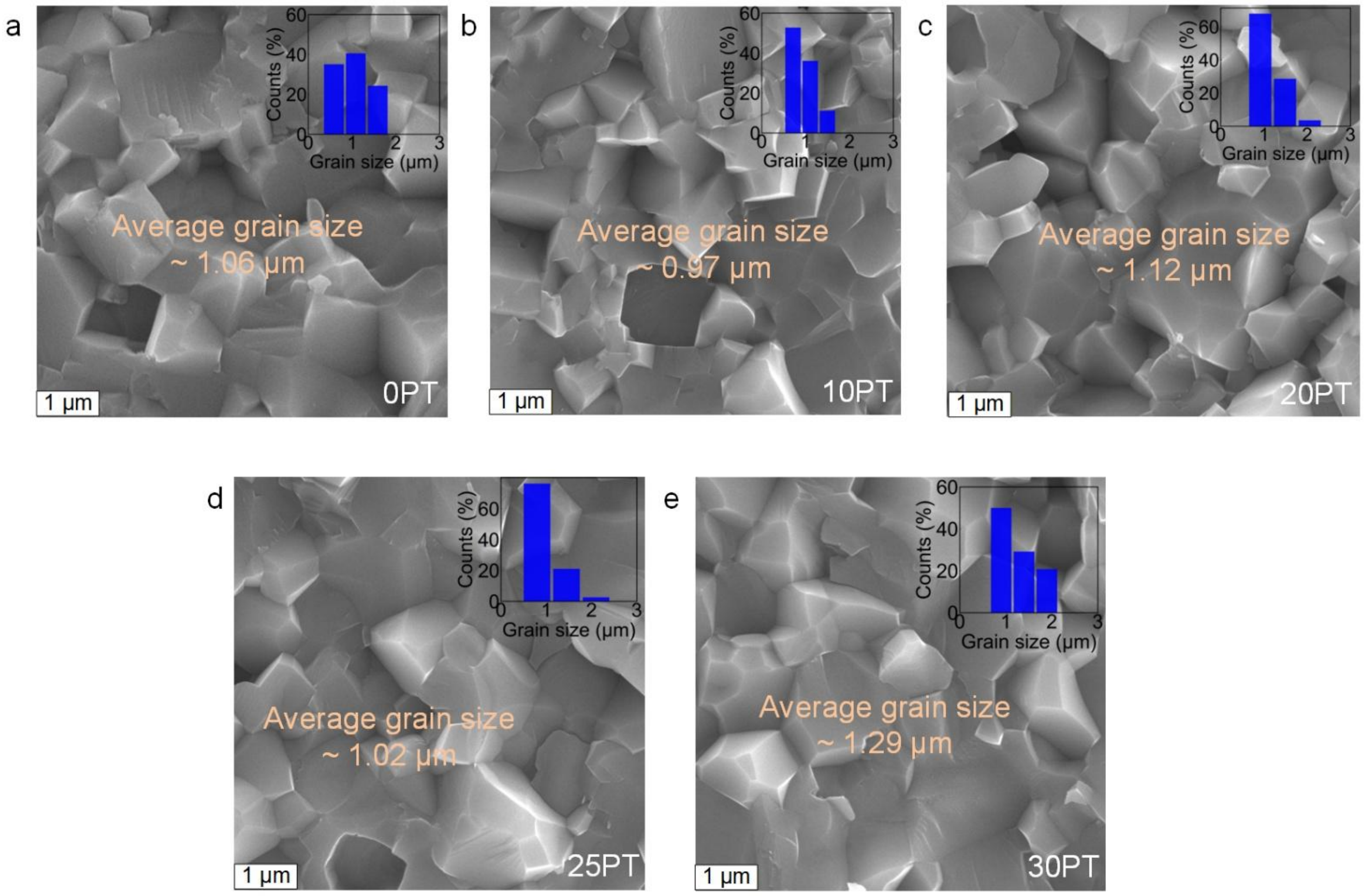

Figure S15 SEM observations of BNKLSTZ-x$PbTiO_3$. (a) x=0; (b) x=0.1; (c) x=0.2; (d) x=0.25; (e) x=0.3

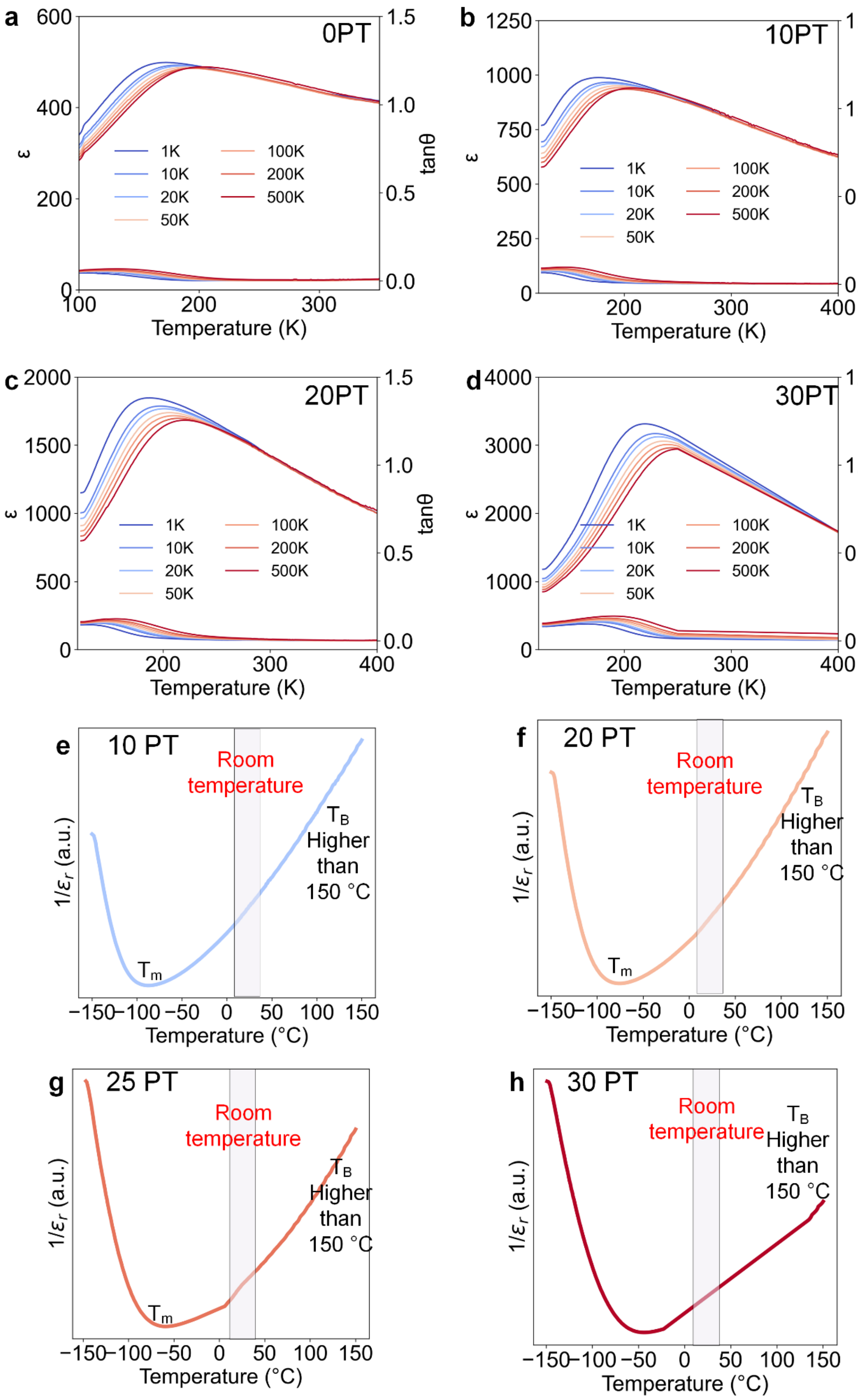

Figure S16. Temperature-dependent permittivity and loss for BNKLSTZ-x$PbTiO_3$: (a) x = 0, (b) x = 0.1, (c) x = 0.2 and (d) x = 0.3; **1/ε_r-*T* of BNKLSTZ-xPT ceramics:** (e)x=0.1, (f)x=0.2, (g)x=0.25 and (h) x=0.3.

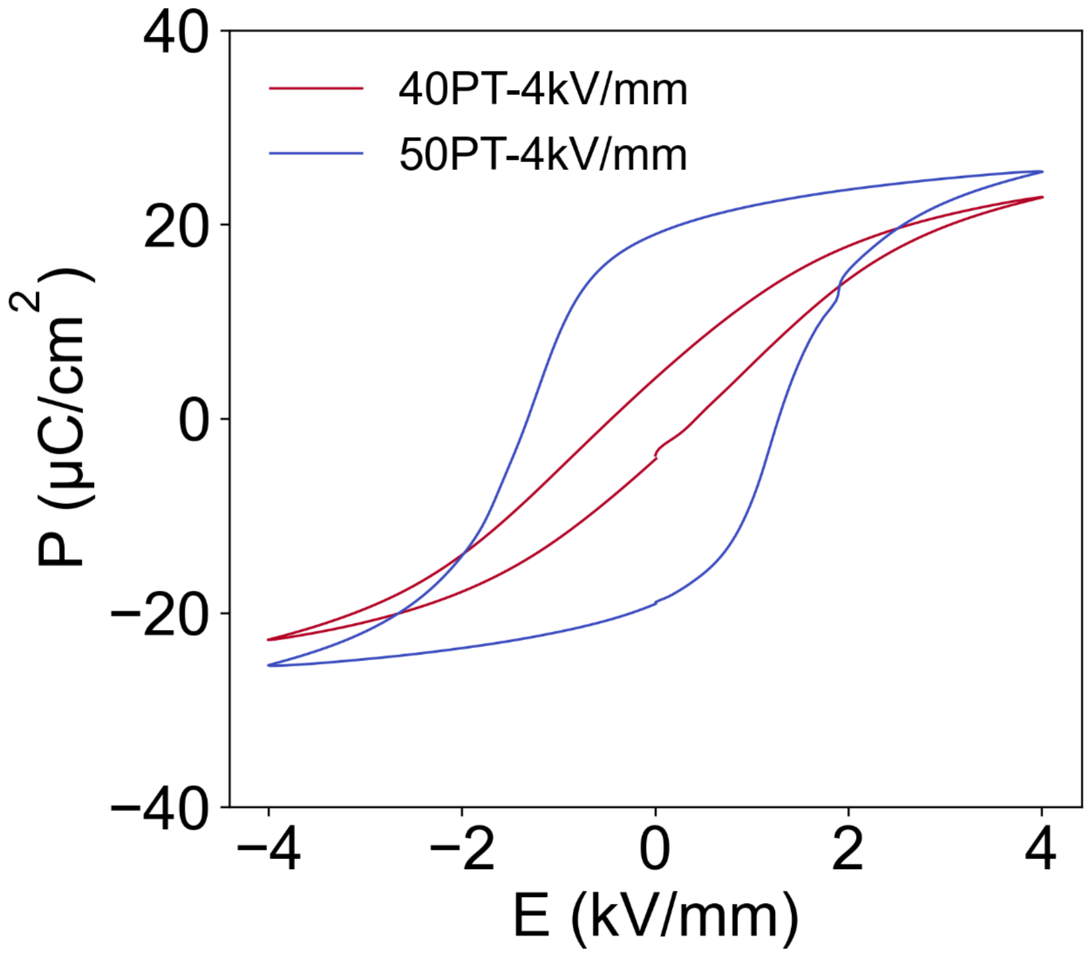

Figure S17 P-E hysteresis loops of BNKLSTZ-based superparaelectric ceramics with different $PbTiO_3$ at 4 kV/mm, x= 40% and 50%.

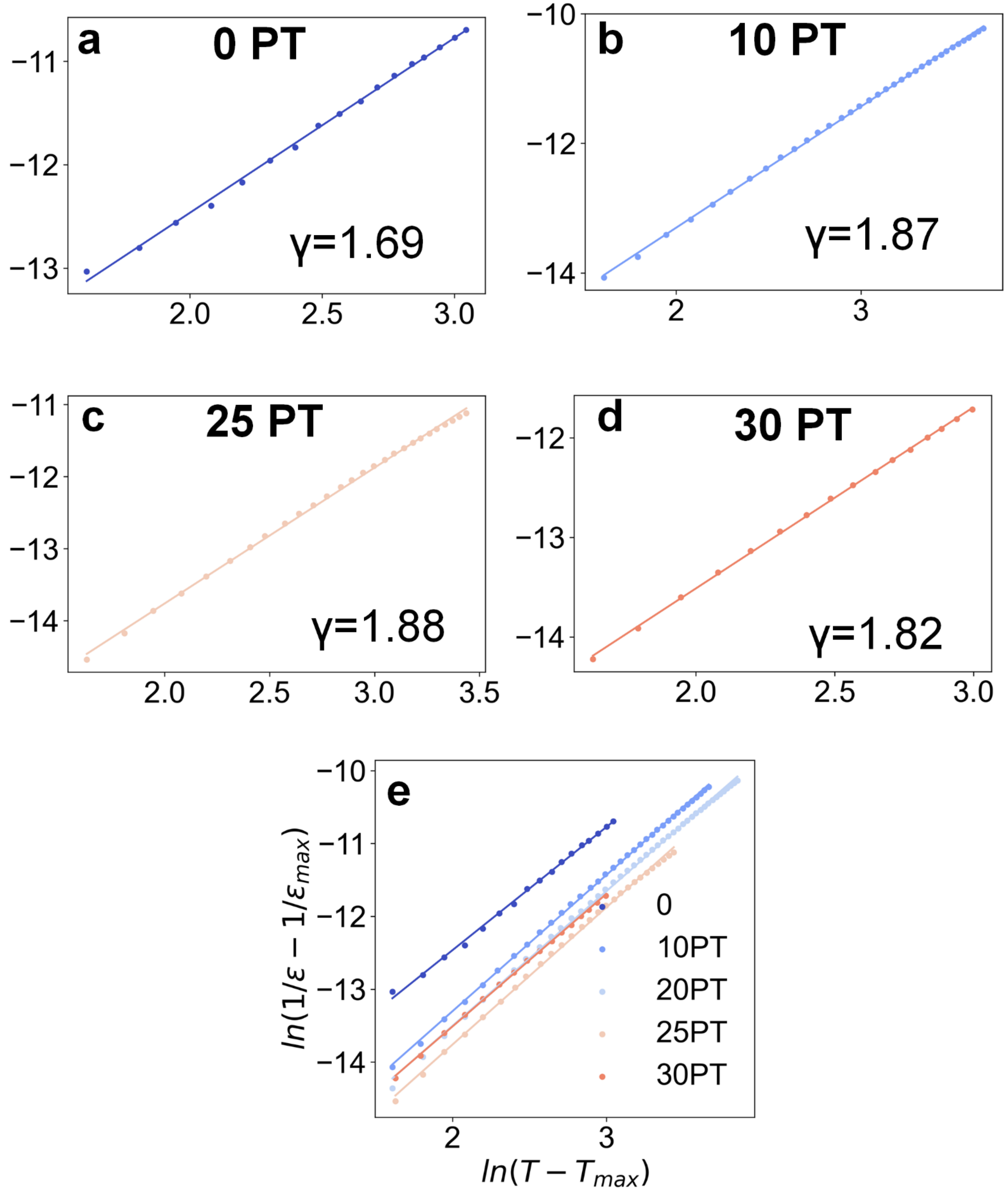

Figure S18 Relaxor degree fitting by modified Curie-Weiss law of BNKLSTZ-based superparaelectric ceramics with different $PbTiO_3$. (a) 0%; (b) 10%; (c) 25%; (d) 30%; (e) Comparison.

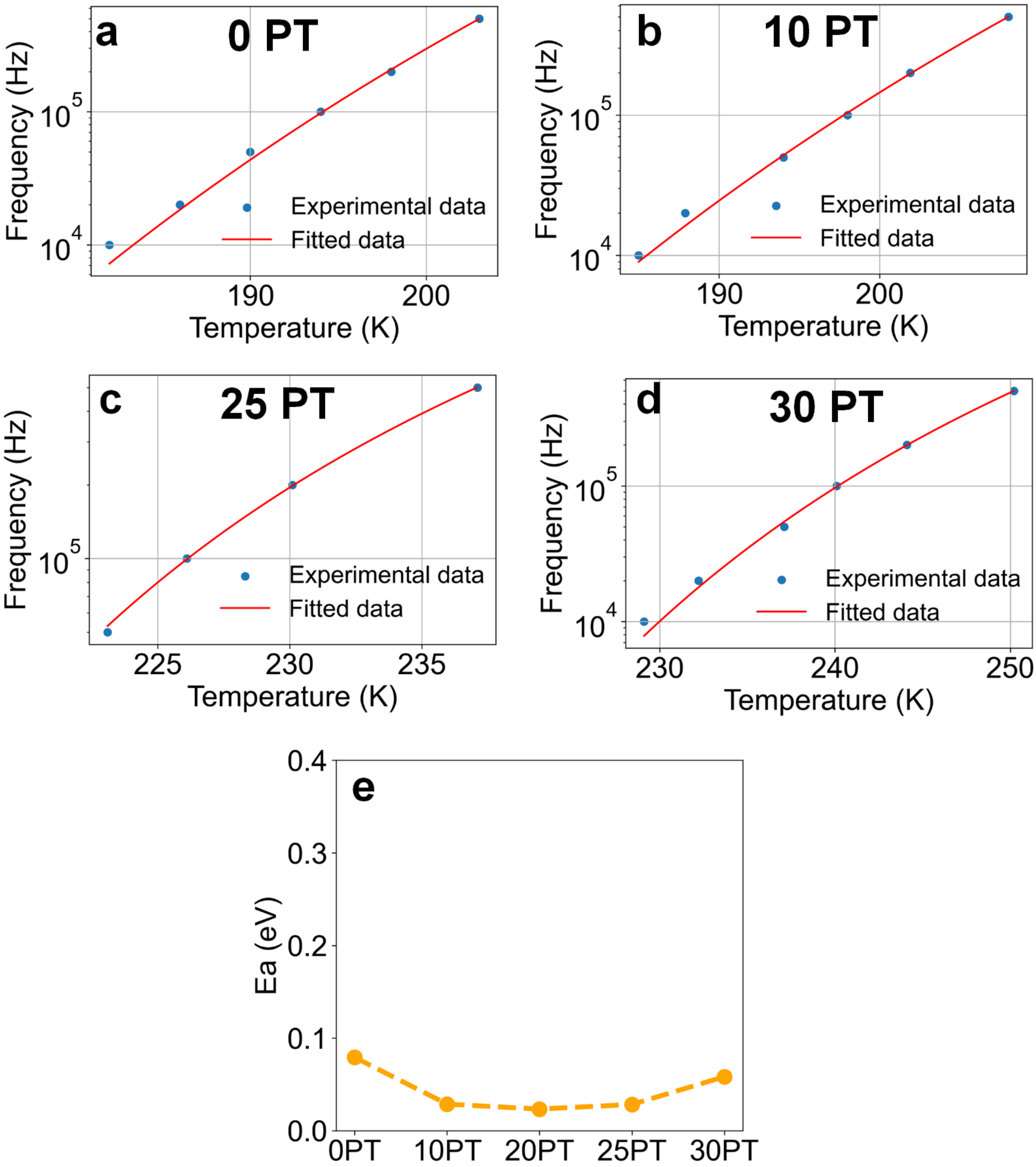

Figure S19 Vogel-Fulcher fitting of BNKLSTZ-based superparaelectric ceramics with different $PbTiO_3$. (a) 0%; (b) 10%; (c) 25%; (d) 30%; (e) Comparison.

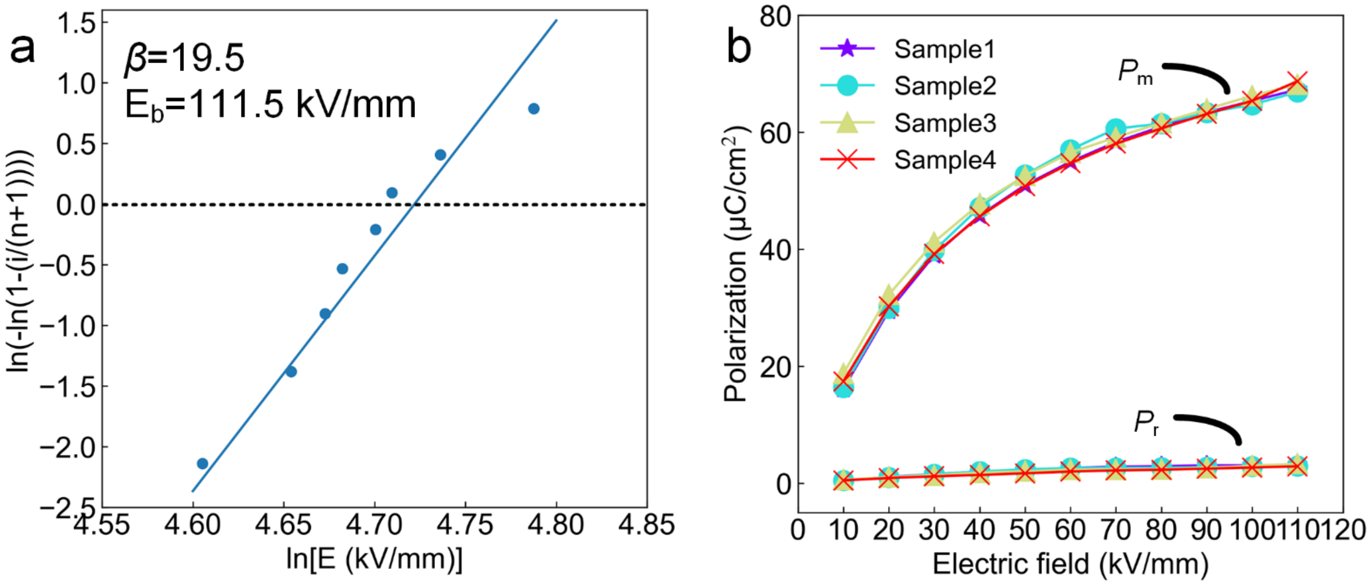

Figure S20 (a) Weibull analysis of the 25PT ceramics; (b) Polarization response as a function of electric field for different 25PT samples.

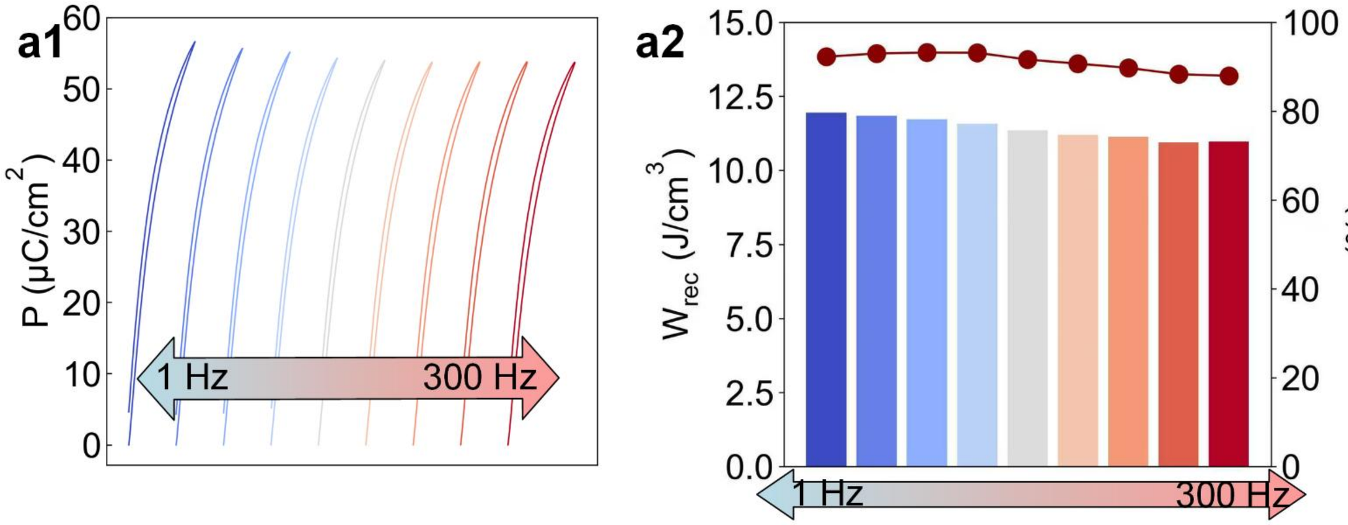

Figure S21 P-E hysteresis loops of the BNKLSTZ-25PT ceramic at 70 kV/mm over a wide range of the frequency from 1 to 300 Hz and the corresponding W_{rec} and η.

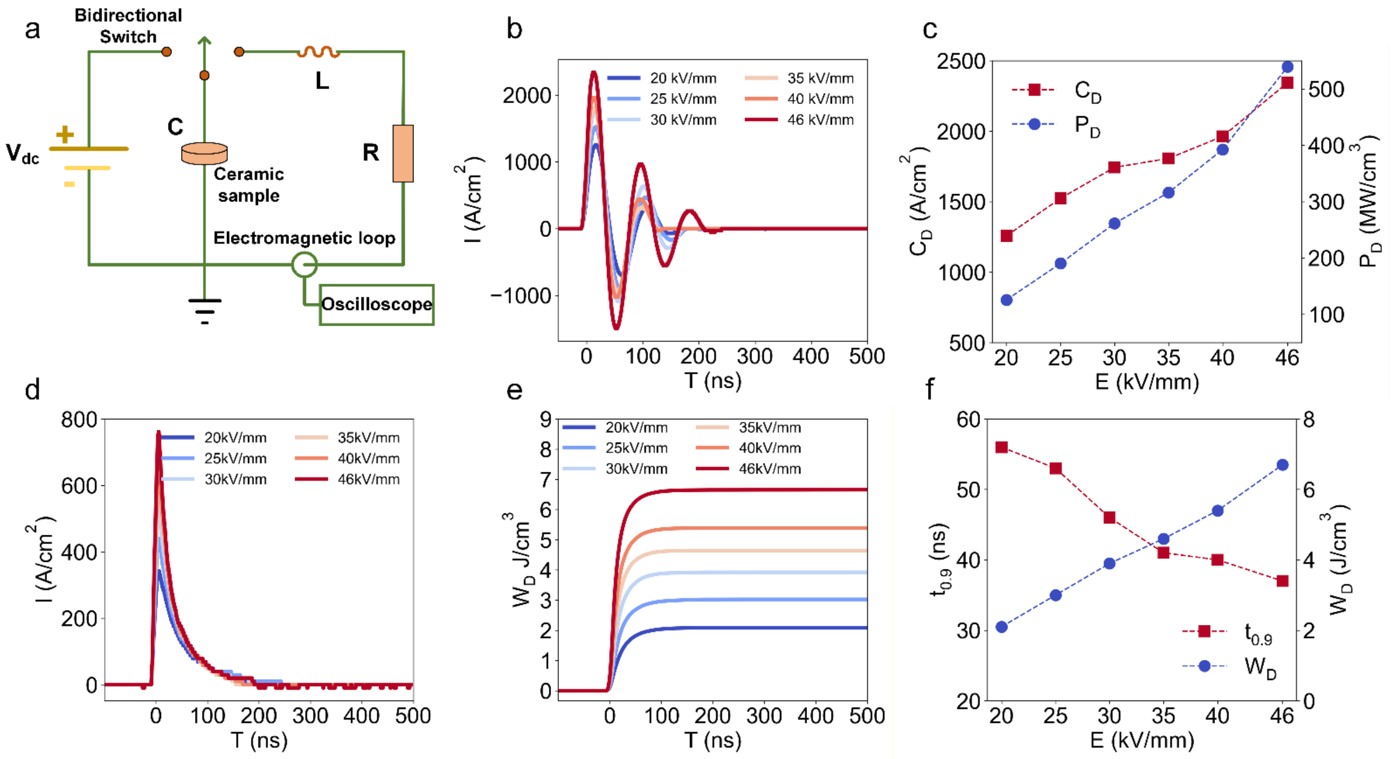

Figure S22 Charge and Discharge Testing (a) Charge and Discharge Testing Circuit Diagram; (b) Under-damped discharge wave forms under different electric fields of the BNKLSTZ-25PT as a function of time; (c) The current density (C_D) and power discharge density (P_D) as a function of applied electric fields; (d) Over-damped discharging current (I-t) curves of the BNKLSTZ-25PT in connection with a fixed load resistance of 300Ω under various electric fields; (e) The discharge energy density (W_D-t) curves under various electric fields; (f) $t_{0.9}$ and W_D under various electric fields.

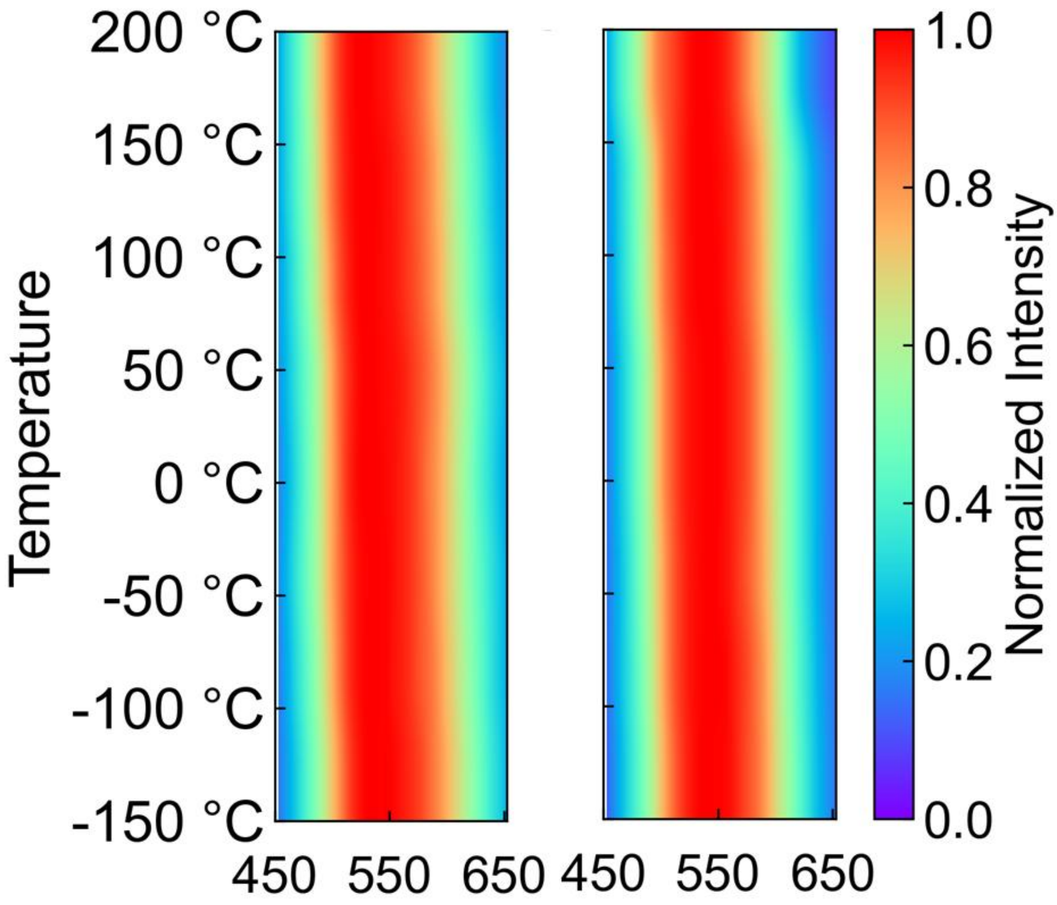

Figure S23 Comparison of Raman spectra for 0PT and 25PT over a broad temperature range. Both ceramics exhibit comparable Raman peak intensities.

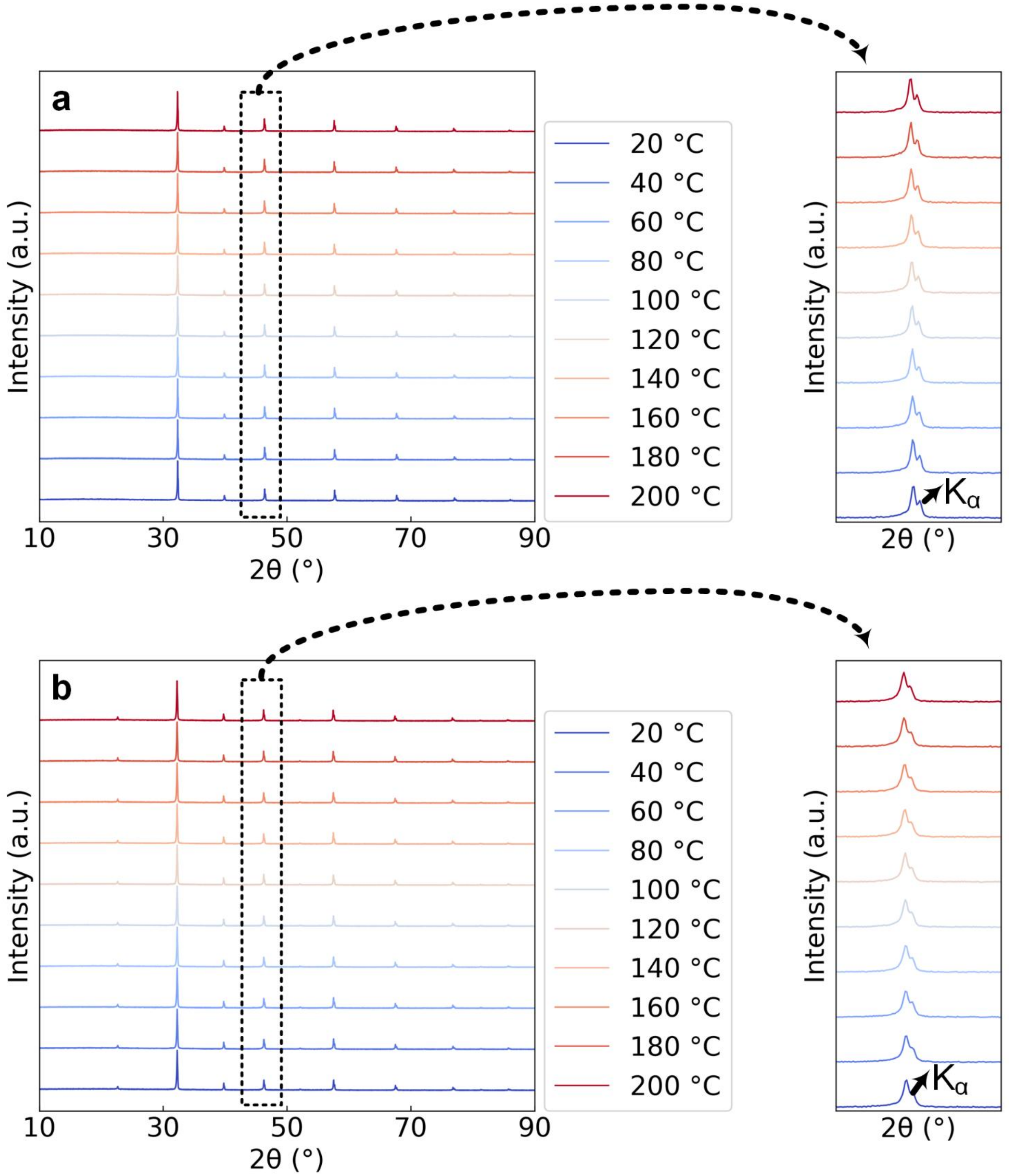

Figure S24 shows the in-situ XRD patterns of BNKLSTZ-x$PbTiO_3$ ceramics. (a) x=0; (b) x=0.25.

Both ceramics exhibit similar average phase structures. For instance, in the main peak region, they display a highly similar single-peak profile, indicative of a pseudocubic phase structure characteristic of a superparaelectric state. Moreover, for the (100) diffraction peak, the x=0.25 ceramic shows a more pronounced intensity, which can be attributed to the enhancement of ferroelectric distortion induced by the introduction of Pb.

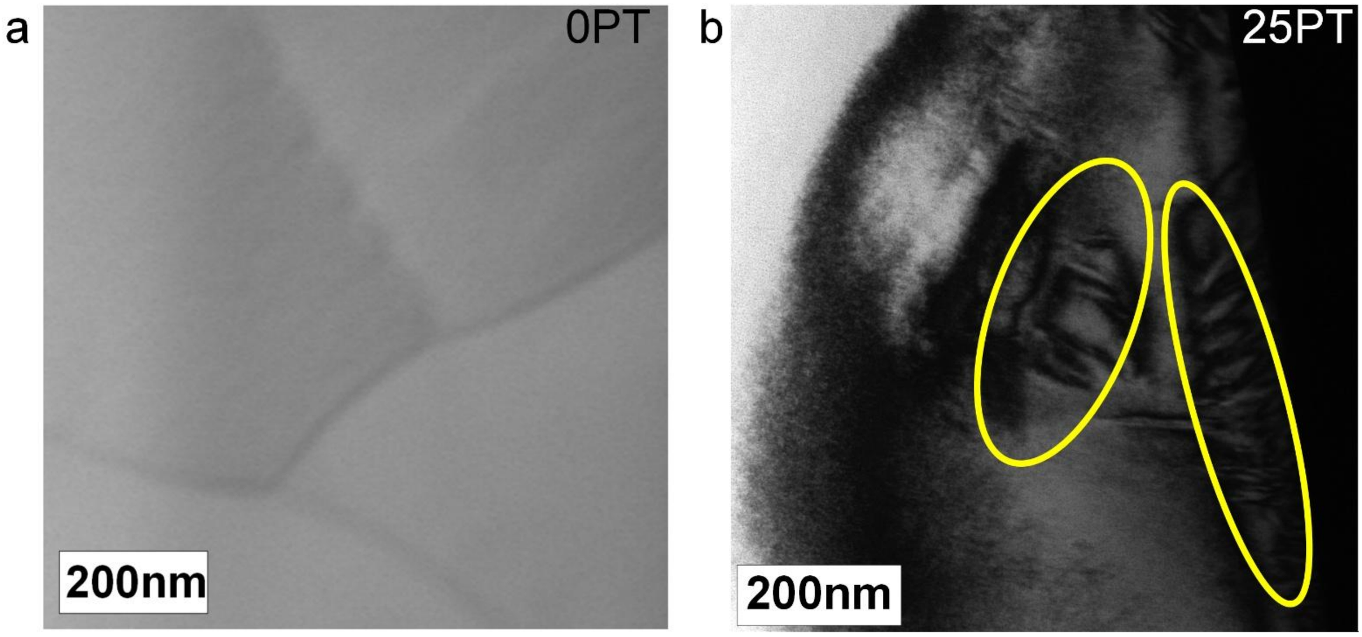

Figure S25 Transmission electron microscopy observation of BNKLSTZ-x$PbTiO_3$. (a) x=0; (b) x=0.25.

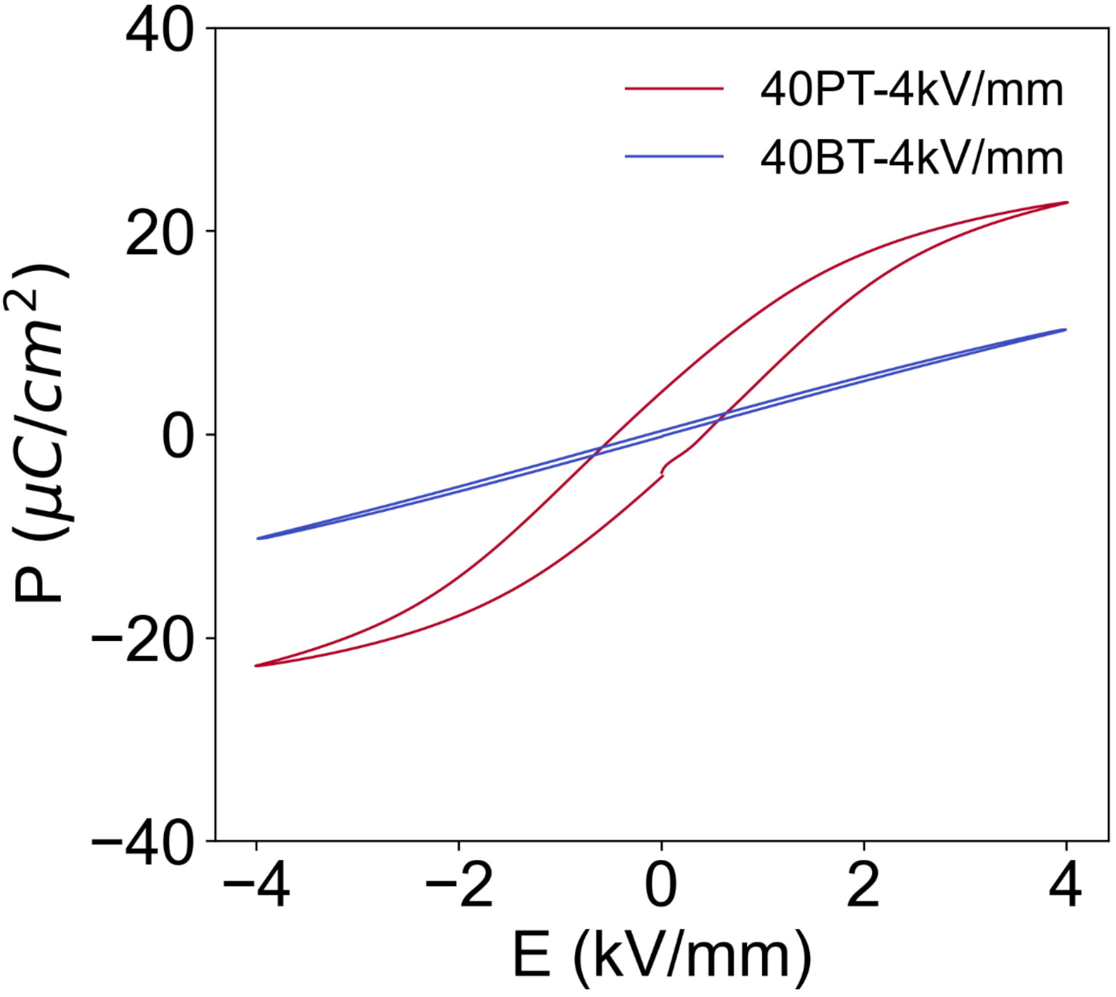

Figure S26 Bipolar ferroelectric property comparison between 40$BaTiO_3$ and 40$PbTiO_3$ modified BNKLSTZ ceramics.

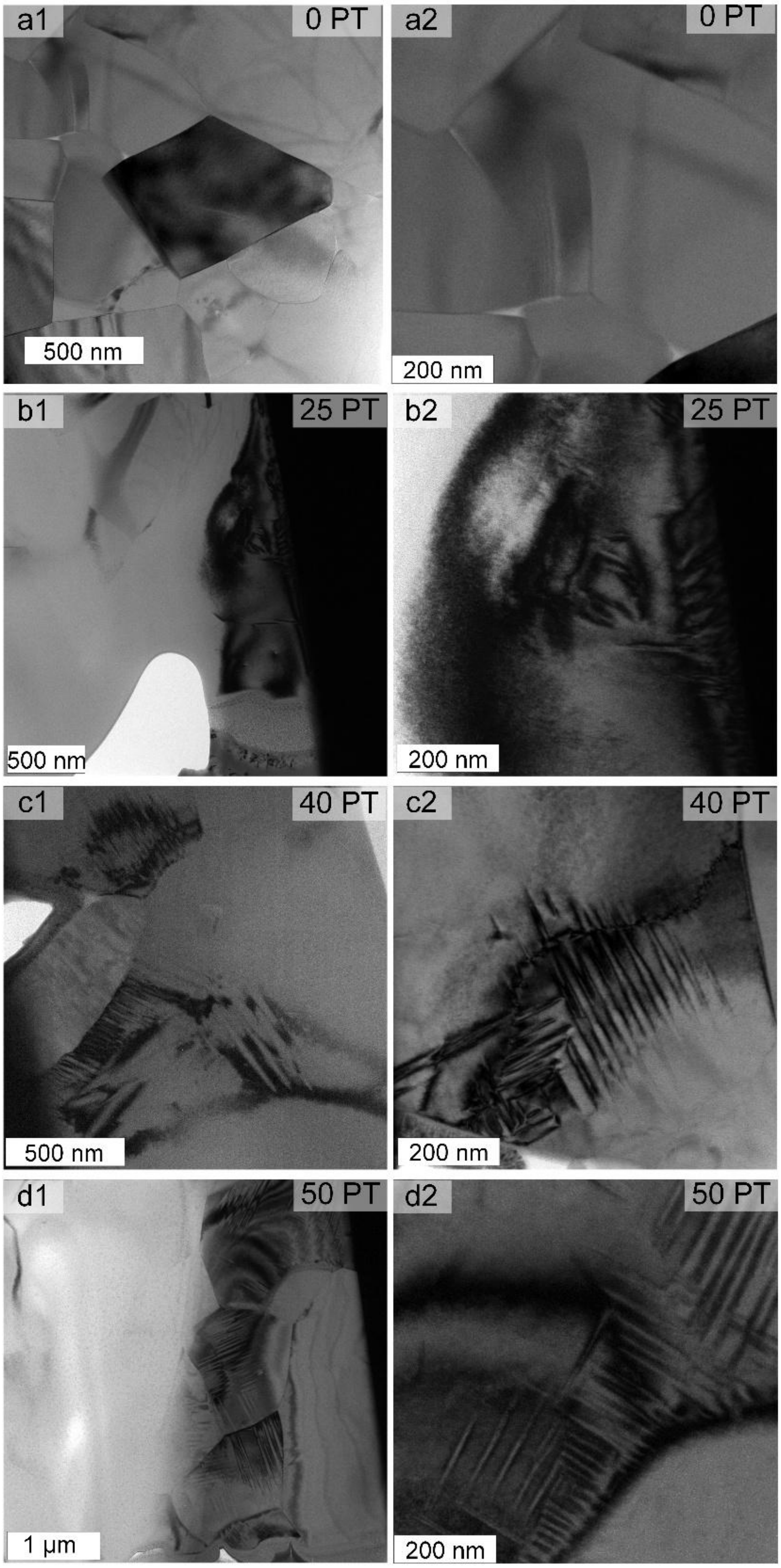

Figure S27 Domain contrast for BNKLSTZ-x$PbTiO_3$. (a) x=0; (b) x=0.25; (c) x=0.40 and (d) x=0.50.

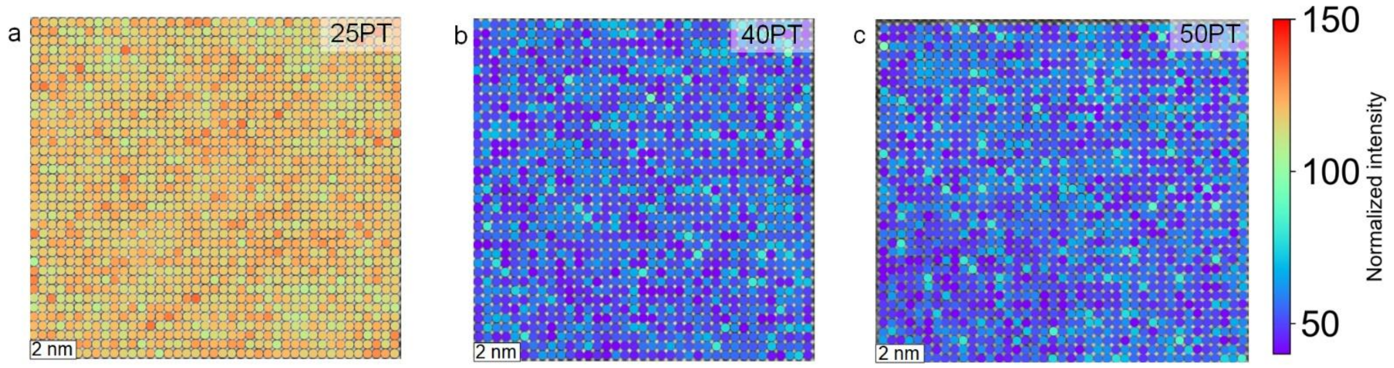

Figure S28 B-site element mapping of the BNKLSTZ-xPbTiO₃. (a) x=0.25; (b) x=0.40 and (c) x=0.50.

With the introduction of more strongly A-site element Pb, the A-site ions appear significantly brighter, leading to a pronounced darkening in the contrast of B-site ions during STEM-HAADF observations.

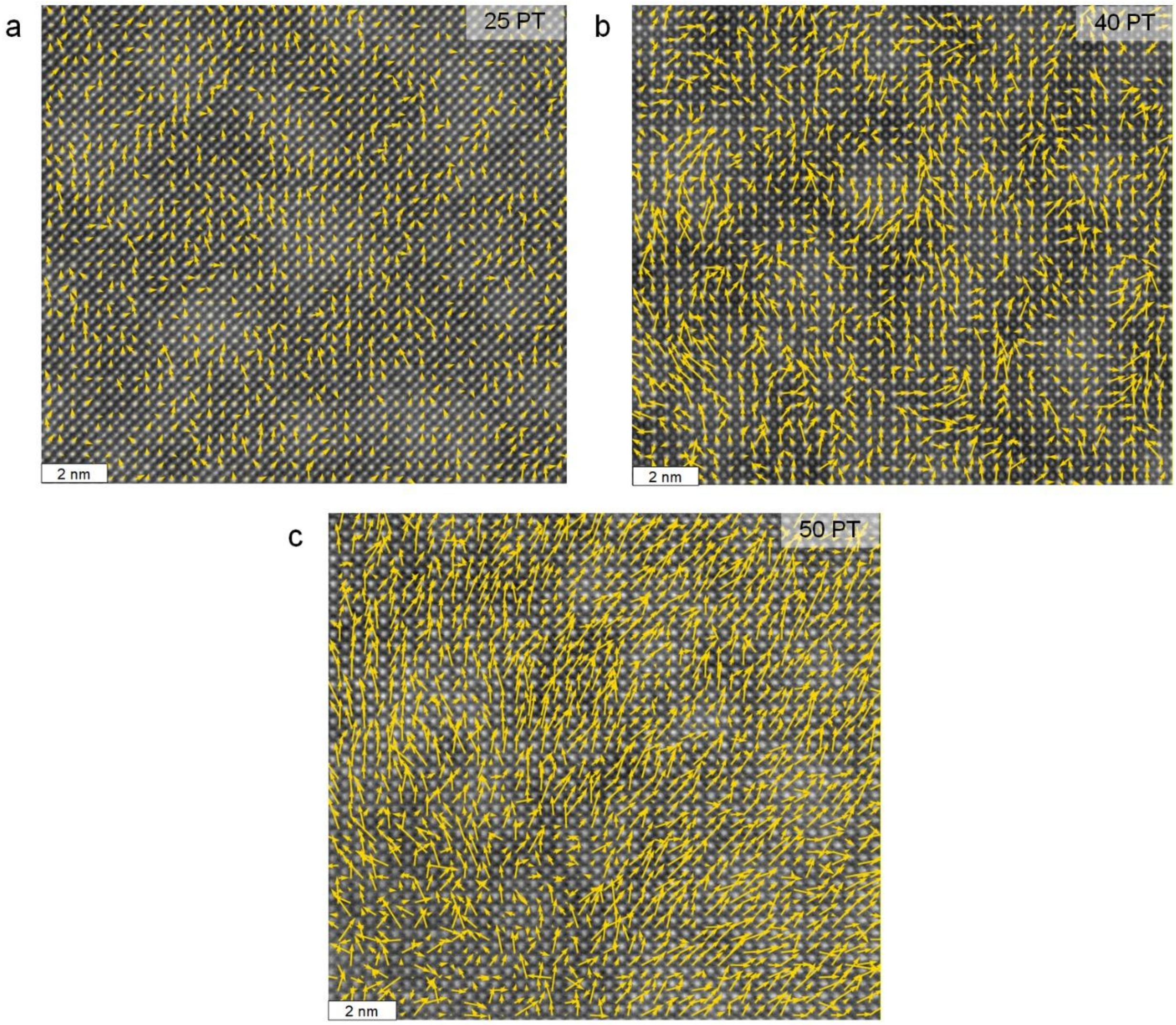

Figure S29 Polarization magnitude along the [001] zone axis of BNKLSTZ-x$PbTiO_3$. (a) x=0.25; (b) x=0.40 and (c) x=0.50.

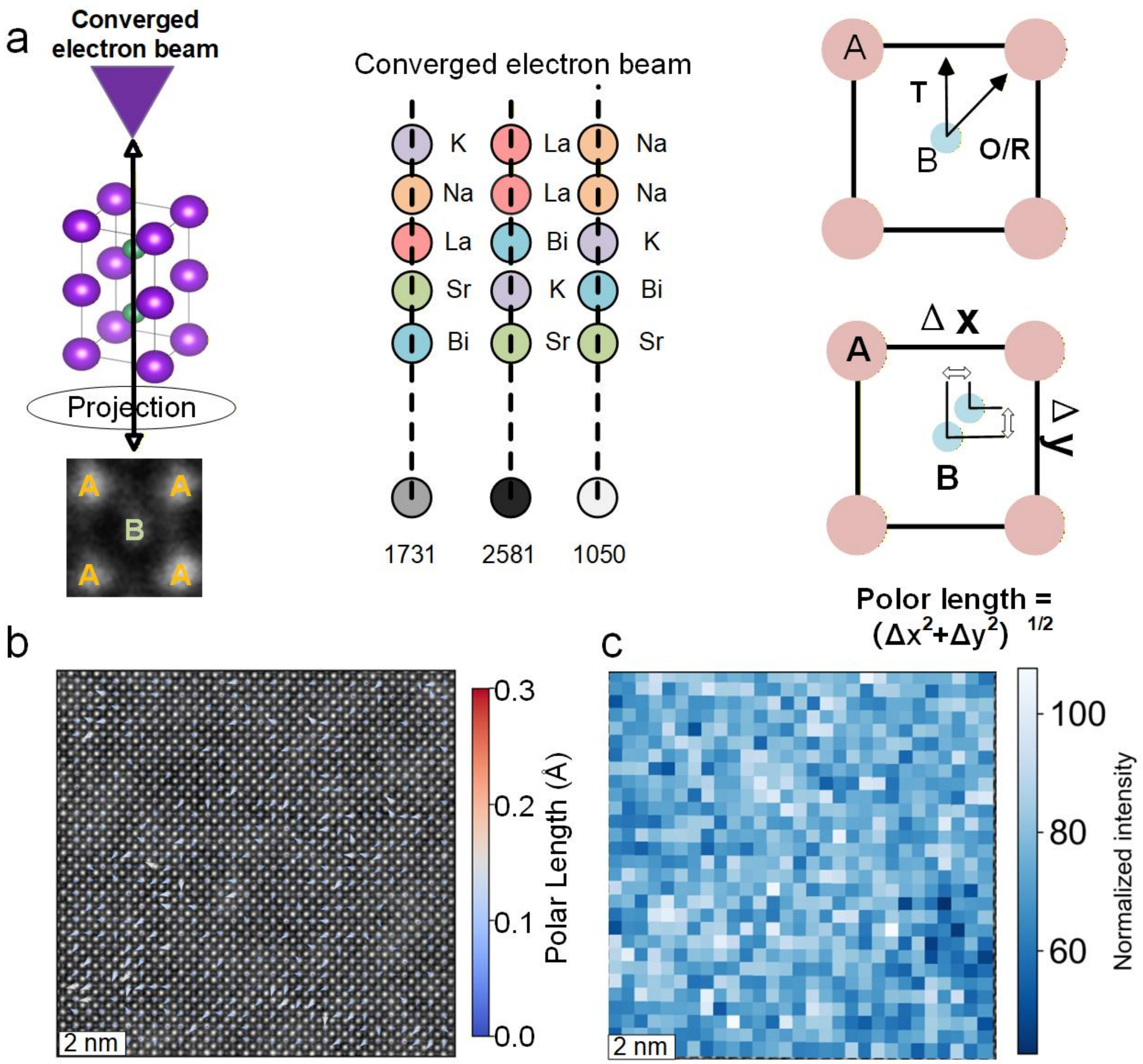

Figure S30 HADDF observation of BNKLSTZ along the [100] zone axis. (a) Illustration of elemental mapping in HADDF image; (b) Polar vector and(c) A-site element mapping.

Supplementary Note 4: DFT calculations for High entropy BNKLST-xPT

DFT calculations were performed by using the Vienna ab-initio simulation package (VASP)[10]. For the exchange-correlation functional, the generalized gradient approximation (GGA) of Perdew-Burke-Emzerhof for solid was used[11]. Electron-ion interactions were described by the projector augmented-wave (PAW) potential with a kinetic energy cut-off of 500 eV. The Pb 5d6s6p, Bi 5d6s6p, Na 2p3s, K 3s3p4s, La 5d6s, Sr 4p5s, Ti 3d4s, and O 2s2p states were treated as valence electrons. The Brillouin zone integrations were sampled by using 1 × 1 × 5 Monkhorst-Pack grids for all the calculations. The energy convergence criterion was set to be 10^{-6} eV and the structure was fully relaxed until the force was <0.02 eV/Å. After the completion of the structural optimization, further calculations of charge density were conducted.

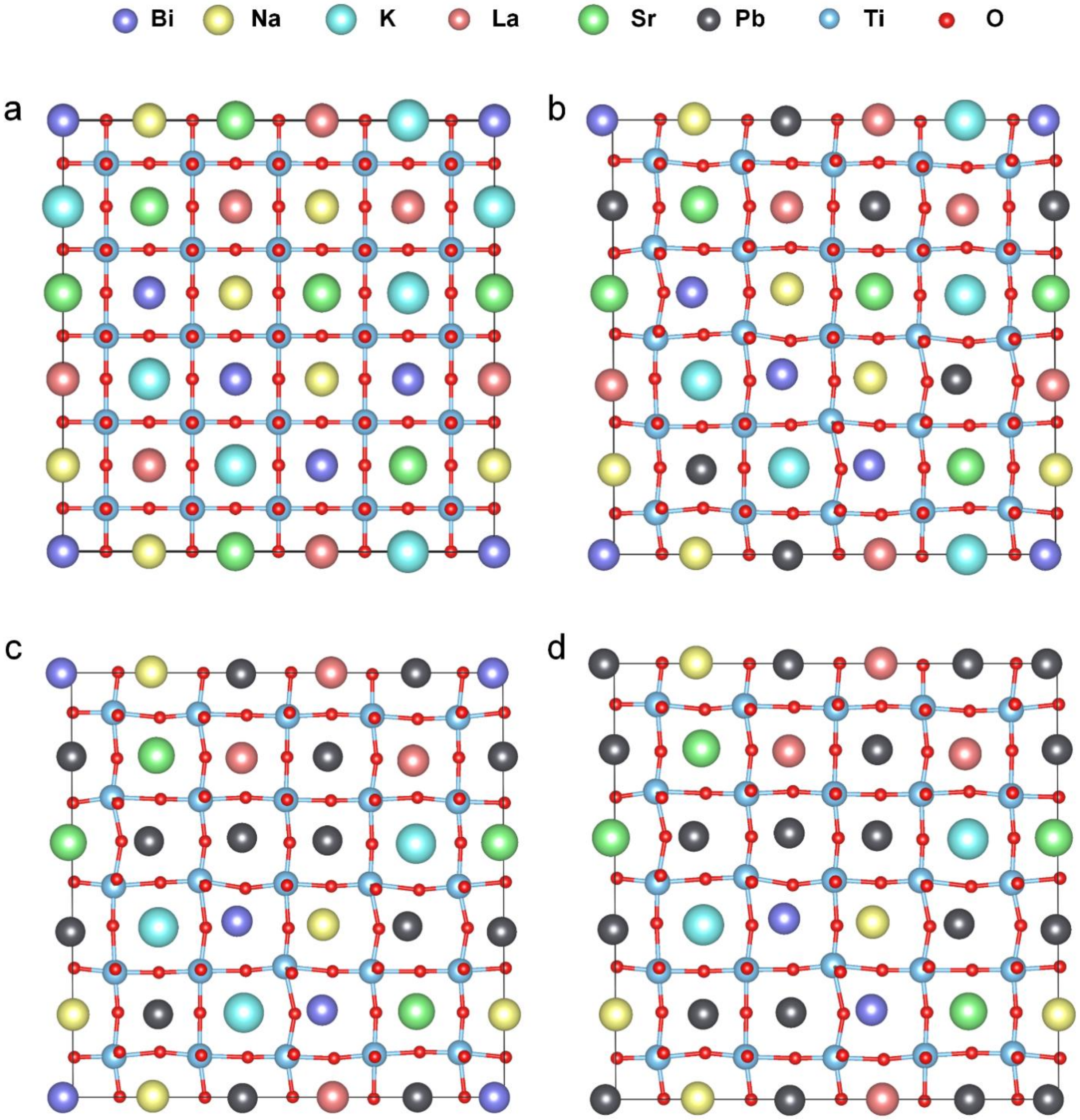

Figure S31 Supercell used for DFT calculations of BNKLST-xPbTiO₃. (a) x=0; (b) x=0.25 (c) x=0.4 and (d) x=0.50.

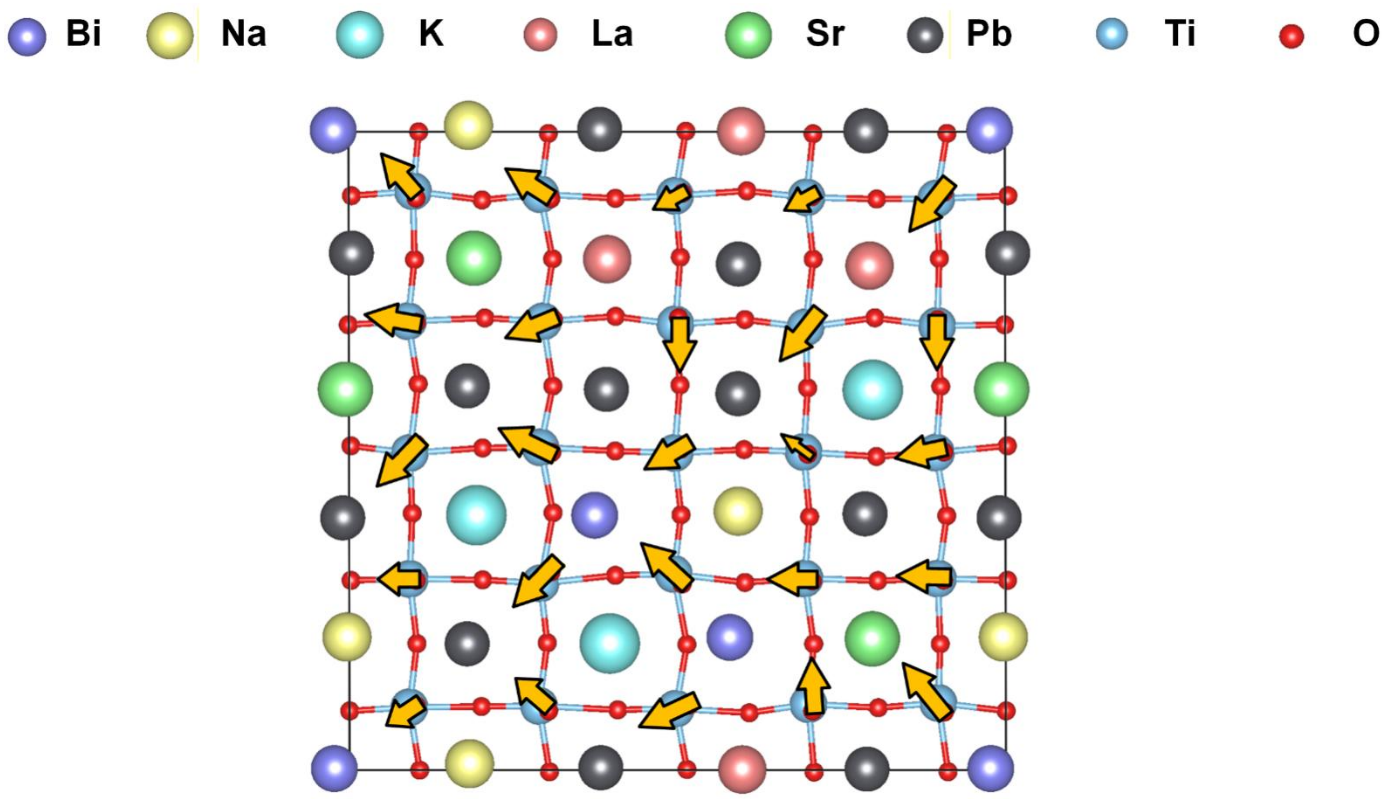

Figure S32 DFT calculation results for the BNKLST-40PbTiO$_3$ ceramic.

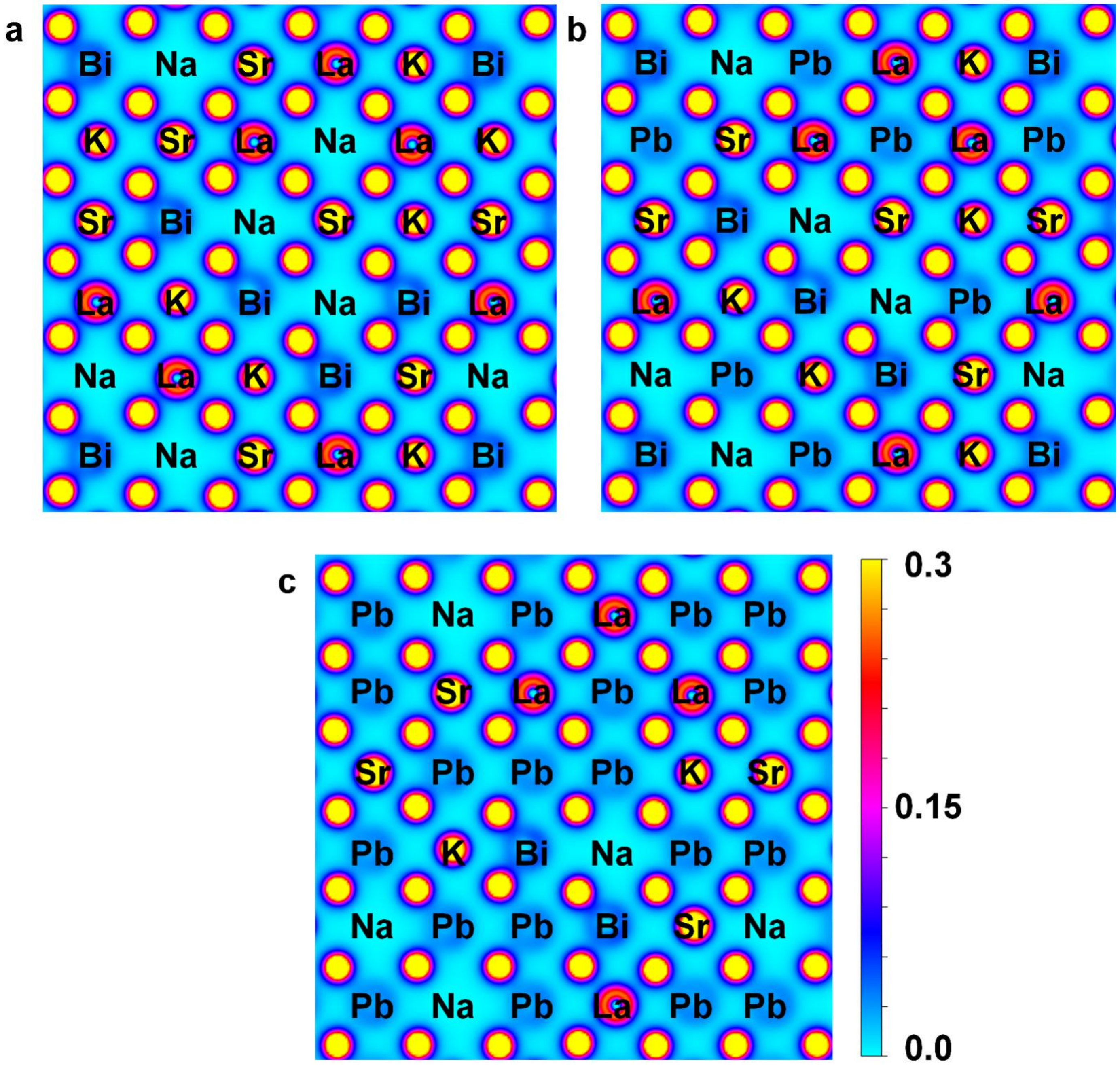

Figure S33 Charge density maps from DFT of BNKLST-x$PbTiO_3$. (a) x=0; (b) x=0.25 and (c) x=0.50.

Supplementary Note 5 Further insights into how the energy storage properties can be potentially enhanced

We have added further insights into how the energy storage properties can be potentially enhanced, based on the mechanisms revealed in our study. Specifically, according to the $W_{rec} = \int_{P_r}^{P_m} E\, dP$, the key to further improving performance lies in the parameters: P_m-P_r, and E.

(1) Enhancing the (P_m−P_r): as shown in Figure S34, we have supplemented additional theoretical calculations to analyze how to further enhance P_m−P_r. Our further phase-field simulations indicate that the critical factor is not merely the introduction of a ferroelectric polarization, but rather the control of its distribution. As illustrated in Figures S34a and c, at a given concentration, a more concentrated distribution of the ferroelectric polarization renders it less susceptible to the influence of the superparaelectric matrix. Consequently, it becomes difficult to maintain a near-zero polarization (large P_r) after the removal of the electric field, thereby deteriorating the energy storage density and efficiency. Conversely, as illustrated in Figures S34b~c, a more dispersed distribution of the ferroelectric polarization enhances the coupling between the ferroelectric and superparaelectric regions, facilitating the return of polarization to its initial state. This leads to a reduced P_r and improved energy storage density and efficiency. Based on the current pressureless sintering process, for the BNKLSTZ-xPT system, the optimal composition lies near x = 0.25. Future work could focus on fine-tuning the composition within the range of x = 0.25 to 0.4, or employing novel sintering techniques to control the size and distribution of ferroelectric polarization. This approach holds the potential to further enhance the (P_m−P_r) value, thereby improving the energy storage density and efficiency.

(2) Enhancing the E_b: as presented in Fig. S20, the current ultrahigh W_{rec} of 21 J/cm^3 is achieved at 110 kV/mm, which is the statistic breakdown field strength. As illustrated in Figs. S35a1-a2, at higher field strengths ~120 kV/mm, the material exhibits an increased energy storage density of ~23 J/cm^3 while maintain efficiency of ~85%. These results indicate that further engineering of the microstructure, such as suppressing elemental volatilization (i.e., Pb), could inhibit electrical trees and enhance E_b, thereby potentially increasing energy storage performances.

(3) Experimental study: Here, as shown in Figs. S35b1, we further employed the double-crucible method to suppress the volatilization of Pb, thereby further enhancing the performance of the 25PT ceramics. As illustrated in Fig. S35b2, at an electric field of 122 kV/mm, both the energy storage density and efficiency of the 25PT ceramics were improved, reaching 24.8 J/cm^3 and 88%, respectively. The suppression of Pb volatilization is likely to have promoted a more uniform distribution of ferroelectric polarization and a denser microstructure, thereby enhancing P_m−P_r and E_b, which ultimately contributed to the improved energy storage performance.

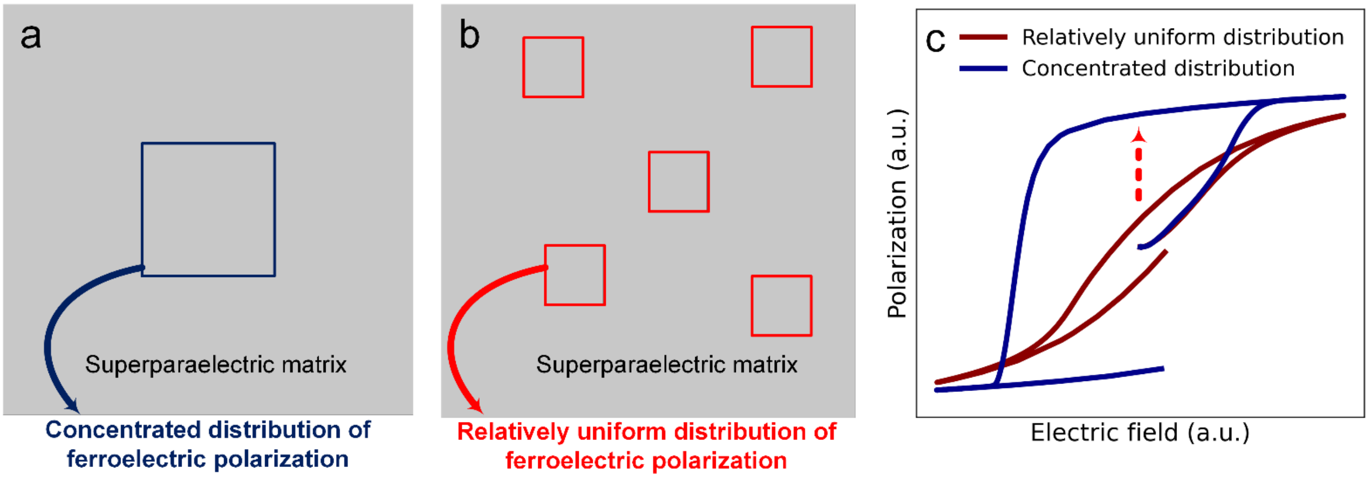

Figure S34 Phase-field simulation on the effects of different ferroelectric polarization distributions on energy storage performance at the same content: (a) Concentrated distribution of ferroelectric polarization; (b) Relatively uniform distribution of ferroelectric polarization; (c) Simulated P-E loops.

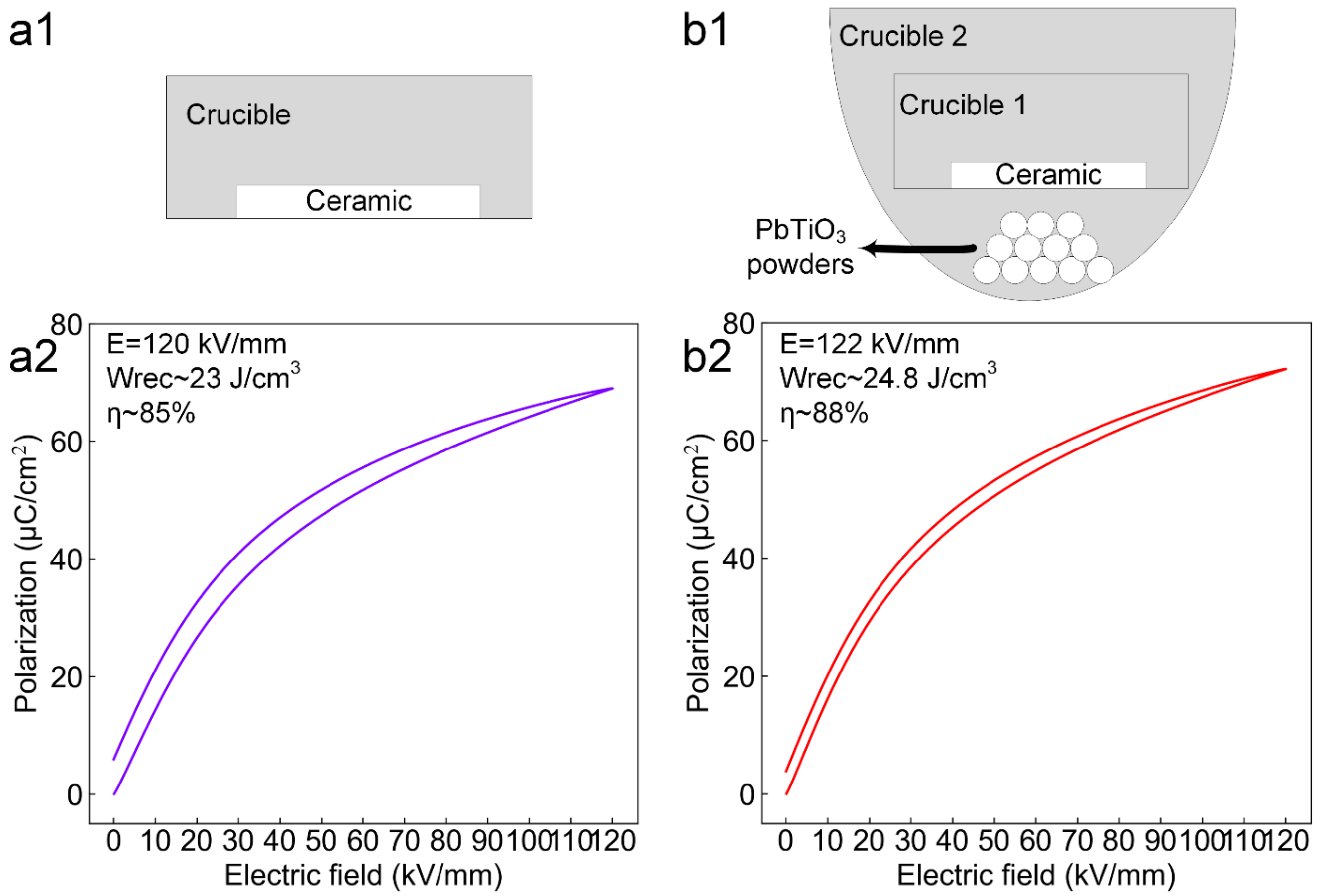

Figure S35 (a) P-E loops under 120 kV/mm of 25 PT ceramics prepared by single-crucible method; (b) P-E loops under 120 kV/mm of 25 PT ceramics prepared by double-crucible method.

References

1 Biner, S. B. *Programming phase-field modeling*. (Springer, 2017).

2 Schrade, D., Mueller, R., Xu, B. X. & Gross, D. Domain evolution in ferroelectric materials: A continuum phase field model and finite element implementation. *Computer Methods in Applied Mechanics and Engineering* **196**, 4365-4374 (2007). https://doi.org/https://doi.org/10.1016/j.cma.2007.05.010

3 Wei, T. *et al.* High-entropy assisted capacitive energy storage in relaxor ferroelectrics by chemical short-range order. *Nat. Commun.* **16**, 807 (2025). https://doi.org/10.1038/s41467-025-56181-6

4 Zeng, S. *et al.* The mechanism for the enhanced piezoelectricity, dielectric property and thermal stability in (K,Na)NbO3 ceramics. *Acta Materialia* **287**, 120801 (2025). https://doi.org/https://doi.org/10.1016/j.actamat.2025.120801

5 Zou, J. *et al.* Unveiling the Origin of Ultrahigh Piezoelectricity in Sb Doped KNN Based Piezoceramics. *Advanced Functional Materials* **n/a**, 2425080 https://doi.org/https://doi.org/10.1002/adfm.202425080

6 Pohlmann, H., Wang, J.-J., Wang, B. & Chen, L.-Q. A thermodynamic potential and the temperature-composition phase diagram for single-crystalline K1-xNaxNbO3 ($0 \le x \le 0.5$). *Applied Physics Letters* **110** (2017). https://doi.org/10.1063/1.4978360

7 Yang, B. *et al.* Engineering relaxors by entropy for high energy storage performance. *Nature Energy* **8**, 956-964 (2023). https://doi.org/10.1038/s41560-023-01300-0

8 Yang, B. *et al.* Enhanced energy storage in antiferroelectrics via antipolar frustration. *Nature* **637**, 1104-1110 (2025). https://doi.org/10.1038/s41586-024-08505-7

9 Haun, M. J., Furman, E., Jang, S. J. & Cross, L. E. Thermodynamic theory of the lead zirconate-titanate solid solution system, part V: Theoretical calculations. *Ferroelectrics* (1989).

10 Kresse, G. & Joubert, D. From ultrasoft pseudopotentials to the projector augmented-wave method. *Physical review b* **59**, 1758 (1999).

11 Perdew, J. P. *et al.* Restoring the density-gradient expansion for exchange in solids and surfaces. *Physical review letters* **100**, 136406 (2008).
